# Unveiling the ecology and spatial dynamics of *Trypanosoma cruzi*, its DTUs and *Triatoma vitticeps* in the Atlantic Forest of south-eastern Espírito Santo State, Brazil

**DOI:** 10.1371/journal.pntd.0014111

**Published:** 2026-03-16

**Authors:** Raphael Testai, Felipe de Oliveira, Maria Augusta Dario, Ane Luíse Quinze Dias de Faro de Oliveira, Flávio Luis de Mello, Ana Maria Jansen, Samanta Cristina das Chagas Xavier

**Affiliations:** 1 Laboratório de Biologia de Tripanosomatídeos, Instituto Oswaldo Cruz, Fiocruz, Rio de Janeiro, Rio de Janeiro, Brasil; 2 Programa de pós-graduação em Biologia Computacional e Sistemas, Instituto Oswaldo Cruz, Fiocruz, Rio de Janeiro, Rio de Janeiro, Brasil; 3 Departamento de Engenheira Eletrônica e Computação, Universidade Federal do Rio de Janeiro, Rio de Janeiro, Rio de Janeiro, Brasil; The University of Tokyo Graduate School of Agricultural and Life Sciences Faculty of Agriculture: Tokyo Daigaku Daigakuin Nogaku Seimei Kagaku Kenkyuka Nogakubu, JAPAN

## Abstract

The transmission dynamics of *Trypanosoma cruzi* in natural environments exhibit considerable variation at the micro-locality scale. However, the specific biotic and abiotic factors driving this heterogeneity remain largely unidentified. The Atlantic Forest of the state of Espírito Santo (ES) presents a unique transmission network of *T. cruzi*, in which *Triatoma vitticeps* represents the absolute majority among existing triatomines, with high infection rates and diversity of genotypes, frequently invading homes. No infection was found in peridomestic mammals. This study aimed to elucidate the spatial and environmental distribution patterns of *T. vitticeps* and its infection by *T. cruzi* DTUs throughout Espírito Santo, quantifying the influence of abiotic variables on both vector occurrence and infection dynamics. Species Distribution Modeling (SDM) of *T. cruzi* genotypes in *T. vitticeps* collected in the Atlantic Forest of Espírito Santo was performed using the ModleR package, in the R programming language, with climate and landscape variables (~1km²) selected by Spearman’s correlation [-0.7 ≤ ρ ≤ 0.7]. True Skill Statistic (≥ 0.7) was used to evaluate model performance. Decision tree to classify *T. vitticeps* infection by *T. cruzi* was created using machine learning algorithms in WEKA 3.8.6 software. The SDMs of *T. vitticeps* and its infection demonstrated: i. Central and South mesoregions presented better environmental conditions for their occurrence; ii. association with mountainous regions with high altitudes, humid and superhumid, with vegetation density and vigor and high values of topographic diversity; iii. Schoener similarity suggests Z3 is mixed, dominated by TcIV and TcIII in Central–South, with TcIII influence Northwest and North Coast; iv. Infection was explained by wind speed, mammal richness, and temperature, with the decision tree identifying 84% of positives and 29% of negatives. *T. vitticeps* may originate in high-altitude regions and disperse via wind to lowlands, promoting domiciliary invasion and supporting previously hypothesized long-distance transmission of *T. cruzi*.

## Author summary

The spatial distribution of *Trypanosoma cruzi* Discrete Typing Units (DTUs) has yet to be clearly associated with specific biomes or host habitats. Niche modeling, used in this study as species distribution modeling (SDM), provided a valuable approach for assessing environmental suitability for the transmission of the main *T. cruzi* genotypes, emerging as a potential predictive tool for identifying areas at risk of transmission in natural environments. This study demonstrates that wind speed functions as an abiotic variable influencing the dispersal capacity of an infected triatomine species (*Triatoma vitticeps*), thereby establishing a novel long-distance transmission mechanism for *T. cruzi*. Spatiotemporal modeling of the parasite–host interaction relies on an empirical environmental assessment of abiotic factors. The findings presented here underscore wind speed as a key abiotic determinant of *T. vitticeps* movement when infected, highlighting its role in enabling long-range *T. cruzi* transmission. In the Atlantic Forest biome of Espírito Santo, particularly within the Central and Southern mesoregions, optimal conditions for such dispersal are associated with the interplay of wind speed, altitude, vegetation density, relative humidity, and topographic diversity. Decision tree analysis further revealed that species richness constitutes a critical biotic variable associated with the infection of triatomines.

## Introduction

Historically, man’s interaction with triatomines must have occurred since their entry into the American continent, as evidenced by the numerous descriptions of human Chagas disease dating back long before European colonization. Cave paintings dated on the walls of rock caves used by prehistoric man in Latin America also support this assertion since there are triatomine species adapted to rocky outcrops [1–3].

*Trypanosoma cruzi* transmission depends primarily on triatomine insects. In addition to the vectorial–contaminative route, in which infective metacyclic forms are released during or shortly after the insect’s blood meal and defecation [4], infection may also occur orally through the predation of infected insects and mammals, as well as through the ingestion of food contaminated with infected triatomines. The oral route is currently responsible for the majority of Chagas disease cases in the country [5–11]. The consumption of raw meat must have been very important among hunter and gatherer groups in prehistory. It is very unlikely that our ancestors cooked the meat of their prey and the oral route is highly favorable to infection. Currently, after the control of *Triatoma infestans* responsible for household transmission, the oral route has once again become the most important route of transmission of *T. cruzi* in Brazil [5–11]. This new/old transmission scenario has resulted in outbreaks of the acute form of the disease, in areas where transmission was previously only enzootic, turning the current epidemiological scenario much more complex than the classical one [12]. Among wild mammals, infection is probably obtained mainly by predation of vertebrate and invertebrate hosts through a complex trophic network, in which each location has its own specificity regarding the members that compose it [5,13].

The great complexity of this new epidemiological scenario lies in the fact that the outbreak areas have almost no features in common, making it necessary to study each outbreak case individually. The only common feature is that populations that are affected by Chagas disease include low socioeconomic status and low conditions of sanitation [12]. Ideally, it would be desirable to carry out these studies under a One Health approach, namely, multisectoral, transdisciplinary, transcultural and, what is of fundamental importance, integrated.

Triatominae (Hemiptera, Reduviidae) are widely distributed throughout the Americas, occurring in many and diverse environments [14,15], which is an indication of the different adaptive processes that these insects have undergone. Their putative association with vertebrate species, which they use as food sources, has never been proven unambiguous, but rather associations with habitats [16–18].

All developmental stages (nymphs and adults) of Triatomine are hematophagous [18], moreover they show a great ability to process different blood sources. Actually, triatomines have huge potential in using alternative food sources as demonstrated by the development of all instars triatomine stages [19] fed on cockroach hemolymph. This dietary flexibility is not accompanied by habitat flexibility as triatomine species demonstrate different adaptabilities to different environments [14,15,17,18,20,21]. Thus, *Triatoma infestans*, a species originating from the Andean highlands, adapted itself to human dwellings and dispersed throughout South America did not adapt, to any wild ecotope – this peculiarity facilitated control actions for this species by that it required one single control measure. Triatomine distribution is focal, according to the extant more suitable habitats [14,22].

*T. vitticeps* is a wild species very common in the forested areas of the Atlantic Forest of Brazil. The species occurs in the Brazilian states of Bahia (BA), Espírito Santo (ES), Minas Gerais (MG), and Rio de Janeiro (RJ) [17]. *T. vitticeps* presents a greater geographic and environmental restriction in the southeast of the Atlantic Forest, in which its distribution is focused and concentrated in the state of Espírito Santo, with areas of environmental suitability focused on the southeast of the Atlantic Rainforest biome [15,23,24]. Wild adult triatomine species, may fly and invade houses attracted by light or in search of blood meals, although they are described as unable to colonize human habitations [25]. This phenomenon is accentuated at the beginning of large-scale human activities such as deforestation [14,26–28].

Most ecological and biological studies of Triatomine were focused on the species of triatomine described as good vectors of *T. cruzi*. The importance as *T. cruzi* vectors is frequently ranked according to the time elapsed between the blood meal and defecation. In this regard, *T. vitticeps* was not considered an efficient vector of the parasite since, under laboratory conditions, this species took more than an hour to defecate, in contrast to *T. infestans*, which eliminated feces in an average of 10 minutes [29–33]. However, in the new epidemiological situation where oral infections prevail, any and all species of triatomine can and should be considered important [4,5,10,11].

A peculiar ecological feature in ES is that human dwelling invasion by *T. vitticeps* occur mainly in locations with irregular relief, in the mountainous regions of the state [34]. Moreover, four *T. cruzi* DTUs (TcI, TcII, TcIII, TcIV) were described as transmitted [11,35]. This enzootic profile is unique and there is no mention in the literature of the simultaneous occurrence of the main *T. cruzi* DTUs in one single species of triatomine. Additionally, other two species of the genus *Trypanosoma* spp. was observed in *T. vitticeps*: *T. c. marinkellei* and *T. dionisii* [11].

The ecological niche of *T. vitticeps* and the spatial distribution of this vector infected by *T. cruzi* have been studied through Ecological Niche Modeling (ENM) [15,23,24], used in this study as Species Distribution Modeling (SDM). This is a technique that uses biotic data on the species and its infection (occurrence points) and associates it with abiotic data (environmental variables) of where these occurrences are located through Machine Learning algorithms, making it possible to outline the Existing Fundamental Niche (“potential niche”), estimating a proportion, at the pixel level (spatial resolution), of the environmental suitability in a given location [23,36]. Several studies have already used ENM to identify the ecological niche of triatomines, seeking to understand areas considered suitable for identifying these vectors [15,23,24,37–40]. In Espírito Santo, this technique may help in understanding the ecology of *T. vitticeps*, providing possible directions for the origin of its infection by *T. cruzi*, something that is treated as a question mark, but which already has hypotheses of origin [10,35,41].

Artificial intelligence (AI) has become a useful approach in different fields, including the biology field [42]. One of the branches of AI, the machine learning (ML) is a system that learns from a database using mathematical algorithms without the necessity to be programmed [43]. The ML essentially attempts to approximate or imitate human abilities to recognize patterns using computation [44]. The application of AI in parasitology has been used through different approaches: i) predictions of clinical manifestations [45]; ii) parasites diagnosis and identification [46–49]; iii) drug study [50] and; iv) environmental approaches to understand the distribution of vector-borne parasites [51,52].

Understanding the spatial and environmental distribution of *T. vitticeps* is essential to elucidate the transmission dynamics of *T. cruzi* in Espírito Santo, clarifying vector mobility, infection sources, and vectorial potential across the landscape. Dario et al. [41] provided valuable contributions by identifying important environmental factors influencing *T. vitticeps* occurrence and infection, such as humidity, temperature, soil type, altitude, and mammalian richness. However, their 10-km buffer-based analyses were restricted to local interpolations, lacking state-wide extrapolation and quantitative assessment of environmental influence ranges.

Building upon these foundations, the present work employs an integrative framework combining species distribution modeling (SDM) and decision-tree classification to predict both vector and infection patterns. Utilizing bioclimatic data from WorldClim and landscape variables from Google Earth Engine (2010–2020), we incorporated novel predictors including wind-speed range and topographic diversity. Furthermore, we modeled the spatial distribution of *T. cruzi* discrete typing units (DTUs) TcII, TcIII, TcIV, and Z3 (TcIII/TcIV), the first such approach in Brazil. By quantifying environmental thresholds and mapping DTU-level spatial patterns, this study advances current knowledge and offers a state-wide perspective on the eco-epidemiology of *T. cruzi* transmission.

## Methods

### Ethical statement

The study was approved by the Secretaria de Estado da Saúde (SESA/ES) from ES state under protocol number: 84029525.

### Study design

We developed six Species Distribution Models (SDMs) for *T. vitticeps* and its infection by *T. cruzi* DTUs to better understand the transmission cycle in the state of Espírito Santo, Brazil, resulting in a total of eight models for this study. We investigated the transmission of *T. cruzi* in the region by integrating the occurrence of *T. vitticeps* infected with *T. cruzi* with climatic variables, landscape characteristics, and host presence. These data were analyzed using decision tree algorithms to identify the variables most associated with the spatial distribution of infected *T. vitticeps*. This approach provides a more comprehensive understanding of the ecological and environmental factors involved in infection dynamics.

### Study area

**Fig 1:**
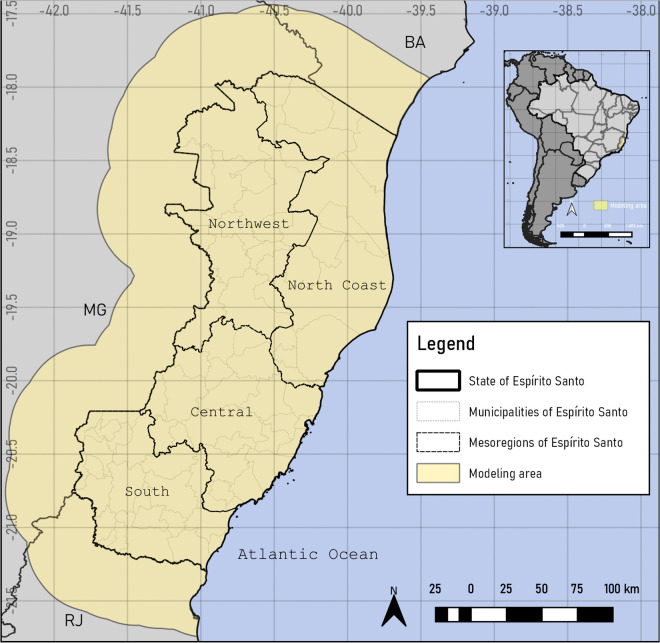
State of Espírito Santo and its mesoregions. Study area of *Triatoma vitticeps* occurrences and their infection by *Trypanosoma cruzi*-like and DTUs, with the Espírito Santo State extended by a 50 km buffer. Software: QGIS 3.22. Source: GADM data version is 4.1. Evaluable from: https://gadm.org/download_country.html.

The study area (Fig 1) was the state of Espírito Santo, in the southeastern region of Brazil. The state is located within the Atlantic Forest biome, with an area of 46,074.448 km² (*https://www.ibge.gov.br/cidades-e-estados/es.html*), bordering Bahia state to the north, the Atlantic Ocean to the east, Rio de Janeiro state to the south, and Minas Gerais state to the west [53]. It is divided into a coastal lowland zone, comprising a strip with altitudes of around 50 m, and a mountainous region, formed by massifs, with altitudes that can reach up to 2,892 m (Pico da Bandeira) [54]. It has a predominantly hot tropical climate, being very humid on the northern coast and in the municipality of Vitória, and mildly mesothermal in the mountainous region. In most of the territory the average temperature is around 18°C, but in the mountainous region it can reach values below 10°C [54,55].

### *Triatoma vitticeps*, *Trypanosoma cruzi-*like and DTUs infection data sources

*T. vitticeps* is reported by locals as frequently found invading local domiciles. When found, residents are advised to capture the insect carefully and take it/them to the nearest Triatomine Information Post (PIT) if available or to contact a municipal health agent for collection [41]. *T. vitticeps* occurrence database (S1 Table) was obtained through the registration of specimens collected in different municipalities and received by the Instituto de Medicina Tropical from the Universidade Federal do Espírito Santo (UFES) and the Laboratório de Biologia de Tripanosomatídeos - LABTRIP (Oswaldo Cruz Institute, Fiocruz) in collaboration with the Núcleo de Entomologia e Malacologia da Secretaria de Estado da Saúde (Nemes/SESA). The triatomines were taxonomically identified according to Lent & Wygodzinsky (1979) and their intestinal content was diluted in saline solution (0.85%) and examined in optical microscopy to observe flagellated forms *T. cruzi-*like. The total number of records in the period 2010–2020 were 2527 specimens: 997 (39.45%) were not infected and 1530 (60.55%) were infected by flagellated forms [35,41]. Among the positive triatomines, 95 DTUs were identified: 36 samples corresponded to DTU TcII, 5 to DTU TcIII, 40 to DTU TcIV, and 14 to Z3. The complementary data obtained from the Nemes/SESA did not include genotype identification, only *Trypanosoma cruzi*-like. To achieve comprehensive coverage across the state, we incorporated infection records from this database (S1 Table).

For the identification of *T. cruzi* DTUs in single and mixed infections a multiplex PCR amplification of the mini-exon gene was performed [56]. It was identified the genotypes TcI (DTU I), TcII (DTU II/V/VI), zymodeme 3 (Z3 - DTU III/IV) and *T. rangeli* species fragments of 200 bp, 250 bp, 150 bp and 100 bp, respectively [57]. To confirm DTU TcII, a PCR-RFLP was performed using 1f8/Alw21I [58]; and for Z3 discrimination, a PCR-RFLP using histone 3 (H3)/AluI [59] was performed to separate the DTUs TcIII and TcIV. Each reaction included negative and positive control samples from *T. cruzi* strains representing the six DTUs. PCR products were visualized in 2% (convencional PCR) and 3% (PCR-RFLP) agarose gel after ethidium bromide staining under ultraviolet light. Mixed infections involving TcII and TcIII/TcIV could not be distinguished, as the PCR-RFLP assay used does not allow discrimination between these two DTUs.

To characterize the environmental conditions associated with the occurrences of *T. vitticeps* and its infection by *T. cruzi* DTUs, we conducted a climatic characterization using the IBGE climate map [55]. In addition, to understand land use and land cover patterns across the state of Espírito Santo, the mesoregions were characterized based on 2023 land use and land cover data (Collection 9) from MapBiomas [60].

### Species Distribution Models: *Triatoma vitticeps*, *Trypanosoma cruzi-*like and DTUs

The SDM were generated using the ModleR package in R [61]. Seven algorithms were used: Maxent, Domain, Mahalanobis Distance, Boosted Regression Trees, Random Forests, Support Vector Machines and the Generalized Linear Models (GLM) statistical method. In addition, Bioclim was used to generate pseudo-absence data. Models were created for: i. *T. vitticeps*; ii. *T. vitticeps* infected with *T. cruzi*; iii. *T. vitticeps* infected with DTU TcII; iv. DTU TcIII; v. DTU TcIV; vi. Zymodeme 3; vii. *T. vitticeps* infected with Z3 and TcIII (Z3/TcIII); and vii. *T. vitticeps* infected with Z3 and TcIV (Z3/TcIV).

For *T. vitticeps*, *T. cruzi*, and the DTUs TcII, TcIV and combination of Z3/TcIV, the k-fold cross-validation partitioning method was used, with 5 partitions and two iterations. To DTU TcIII, Z3 and combination of Z3/TcIII modeling, due to the number of points, the k-fold cross-validation by jackknife was applied with 5 partitions, 14 partitions and 18 partitions, respectively, all in two iterations. The k-fold cross-validation method was applied using five partitions to balance the proportion of test (20%) and training (80%) occurrences, minimizing bias caused by the limited number of training samples due to the small DTU dataset [62]. In the jackknife approach, training data are prioritized over testing data, allowing for improved model performance when dealing with a highly reduced number of occurrences [62].

All the algorithms were processed in the modeling of the *T. vitticeps*, *T. cruzi* and combination of Z3/TcIV. Due to the low number occurrences, the algorithms Mahalanobis Distance to TcII, TcIV, Z3 and combination of Z3/TcIII, and Boosted Regression Trees to Z3 and combination of Z3/TcIII, were not performed.

The evaluation of the models was carried out through the TSS (True Skill Statistics), being used as a cut-off threshold to define the selection of models with good results TSS values ≥ 0.7. The TSS is a metric derived from the confusion matrix that relates the sensitivity, specificity, and accuracy of predicted presences and absences, and is defined as sensitivity + specificity – 1, being considered an appropriate technique for species distribution modeling, particularly for presence–absence maps [63]. The ensemble models for *T. vitticeps*, its infection by *T. cruzi*, and the DTUs were generated by calculating the mean pixel value across all partitions that met the established statistical quality criteria (TSS ≥ 0.7), resulting in one ensemble model for each modeling approach.

Habitat suitability was classified as low (0–33%), medium (33–66%), and high (66–100%). To assess environmental and geographical similarity among models of *T. vitticeps*, *T. cruzi*, DTUs and Z3 (including Z3/TcIII and Z3/TcIV), Schoener’s D index was calculated for all pairwise comparisons.

### Approaches spatial and geographic to analyze the species’ distribution data

Spatial and geographic filters were applied to minimize the influence of hard-to-measure variables and systematic errors, such as bias from sampling effort and the resulting overrepresentation of occurrences or environmental conditions in specific areas. The database was filtered by: (i) removing duplicate coordinates; (ii) keeping only one occurrence per pixel; (iii) excluding triatomine records in pixels without data; and (iv) applying a geographic filter.

The geographic filter created a 5 × 5 km grid across the study area, retaining a single point per cell. This approach is similar to the “uniqueness per pixel” filter, but based on a coarser 25 km² resolution. For *T. cruzi* and its DTU datasets, this filter was not applied due to the small sample size and lack of spatial clustering.

Two types of algorithms were used: i) presence-only and; ii) presence and absence. The latter require the generation of pseudo-absences in regions with low or no environmental suitability. One thousand pseudo-absences were generated between the inclusion (β) and exclusion (α) buffers for each algorithm, where β defines the maximum sampling limit and α excludes areas near presence points. The pseudo-absence points were generated within the β–α buffer zone [64,65]. In the case of Maxent (presence-only), in addition to the 1,000 pseudo-absences created for TSS analysis, the algorithm generated 10,000 background points by default during the modeling process. Inclusion buffers were generated considering the median distance between each occurrence: *T. vitticeps* (82.92 km); *T. cruzi* (61.25 km); and the DTUs TcII (48.5 km), TcIII (36 km), TcIV (49.54 km); Z3 (33.12 km). Exclusion buffers were set within a ~ 10 km radius of each occurrence.

To define cutoff values for pseudo-absence selection, an environmental envelope (Bioclim) approach, constructing a multidimensional range of minimum and maximum environmental values. For *T. vitticeps*, which had 299 occurrences, areas with suitability <10% were considered absences. For *T. cruzi*, DTUs (TcII, TcIII, TcIV) and Z3 (alone or in combination with TcIII/TcIV), areas up to 90% suitability were classified as potential presence zones, and pseudo-absences were generated from pixels below the top 10% suitability.

### Environmental predictors

The bioclimatic and the elevation variables were obtained from the WorldClim database (Table 1) at 30 arc-seconds resolution (~1km²), and the landscape variables obtained from the Google Earth Engine platform (https://earthengine.google.com/), resampled to 30 arc-seconds (~1km²). The Normalized Difference Vegetation Index (NDVI; S1 Appendix) was generated by processing MODIS Terra satellite images, combining the near-infrared and red (visible) bands, originally at a 926.625 m resolution, and resampled to 30 arc-seconds (~1 km²) using the nearest neighbor method. NDVI values represent the median of images collected between 01/01/2010 and 03/01/2020, covering the study period. The SRTM Topographic Diversity (S2 Appendix), representing variation in temperature and humidity conditions as local habitats, was acquired at 270 m resolution and resampled to 30 arc-second (~ 1 km²).

**Table 1:** Name of the 19 bioclimatic variables available on the WorldClim (https://www.worldclim.org/) version 2.1 climate data for 1970-2000. Bioclimatic variables derived from monthly temperature and precipitation data, representing annual trends, seasonality, and extreme environmental conditions.

Bioclimatic variables	
BIO 1: Annual Mean Temperature	BIO 11: Mean Temperature of Coldest Quarter
BIO 2: Mean Diurnal Range (Mean of monthly (max temp - min temp))	BIO 12: Annual Precipitation
BIO 3: Isothermality (BIO2/BIO7) (×100)	BIO 13: Precipitation of Wettest Month
BIO 4: Temperature Seasonality (standard deviation ×100)	BIO 14: Precipitation of Driest Month
BIO 5: Max Temperature of Warmest Month	BIO 15: Precipitation Seasonality (Coefficient of Variation)
BIO 6: Min Temperature of Coldest Month	BIO 16: Precipitation of Wettest Quarter
BIO 7: Temperature Annual Range (BIO5-BIO6)	BIO 17: Precipitation of Driest Quarter
BIO 8: Mean Temperature of Wettest Quarter	BIO 18: Precipitation of Warmest Quarter
BIO 9: Mean Temperature of Driest Quarter	BIO 19: Precipitation of Coldest Quarter
BIO 10: Mean Temperature of Warmest Quarter	

As the Wind Speed and Water Vapor Pressure variables were available from Worldclim with monthly values, they were transformed to calculate the annual maximum and minimum per pixel (max – min). Spearman’s correlation were computed for all possible combinations of environmental variables to select a subset with correlation values between -0.7 and +0.7. The correlation analysis was performed using the R Stats package (version 3.6.2).

### Classification by Decision Tree: *Triatoma vitticeps* and *Trypanosoma cruzi*-like

The database was compiled to perform decision tree classification using biotic and abiotic variables encoded numerically. The response variable was the occurrence of *T. vitticeps*, infected or uninfected, and the following covariables were included: locations and month of capture, NDVI, climatic and landscape variables obtained from the WorldClim database (precipitation, wind speed, thermal amplitude, minimum and maximum temperature, vapor pressure, altitude) and mammal species richness, that were estimated in the software ArcGIS v. 9.3 (ESRI, Redlands, CA, EUA) for each mesoregion of Espírito Santo. The mammal occurrence data was obtained from the Global Biodiversity Information Facility (GBIF; http://doi.org/10.15468/dl.dootzs) and Sistema de Informação Ambiental do Biota (SinBiota; https://sinbiota.biota.org.br/) databases. Each model represented a combination of covariates potentially influencing the probability of *T. vitticeps* infection.

The mammal species richness variable was generated by constructing convex polygons, with the vertices of each polygon corresponding to the occurrence points of each species [41]. Species richness was quantified as the number of individuals within the intersections of these polygons, defined by area rather than by pixel. This variable was not included in the SDM due to its data structure, which was based on area-level estimates limited to the surroundings of *T. vitticeps* occurrence and infection sites. Consequently, it did not provide a continuous, pixel-level spatial representation across the state of Espírito Santo.

Analyses were performed in WEKA 3.8.6 [66], applying J48 [67], REPTree and Logistic Model Tree (LMT) [68] algorithms, which provide a structured explanations of classification criteria. Models were trained using cross-validation, and their performance was assessed using sensitivity, specificity, true positive (TP), false positive (FP), true negative (TN), false negative (FN), precision, recall, F-score, and AUC (area under the ROC curve).

## Results

### Selection of environmental covariables for distribution modeling

**Fig 2:**
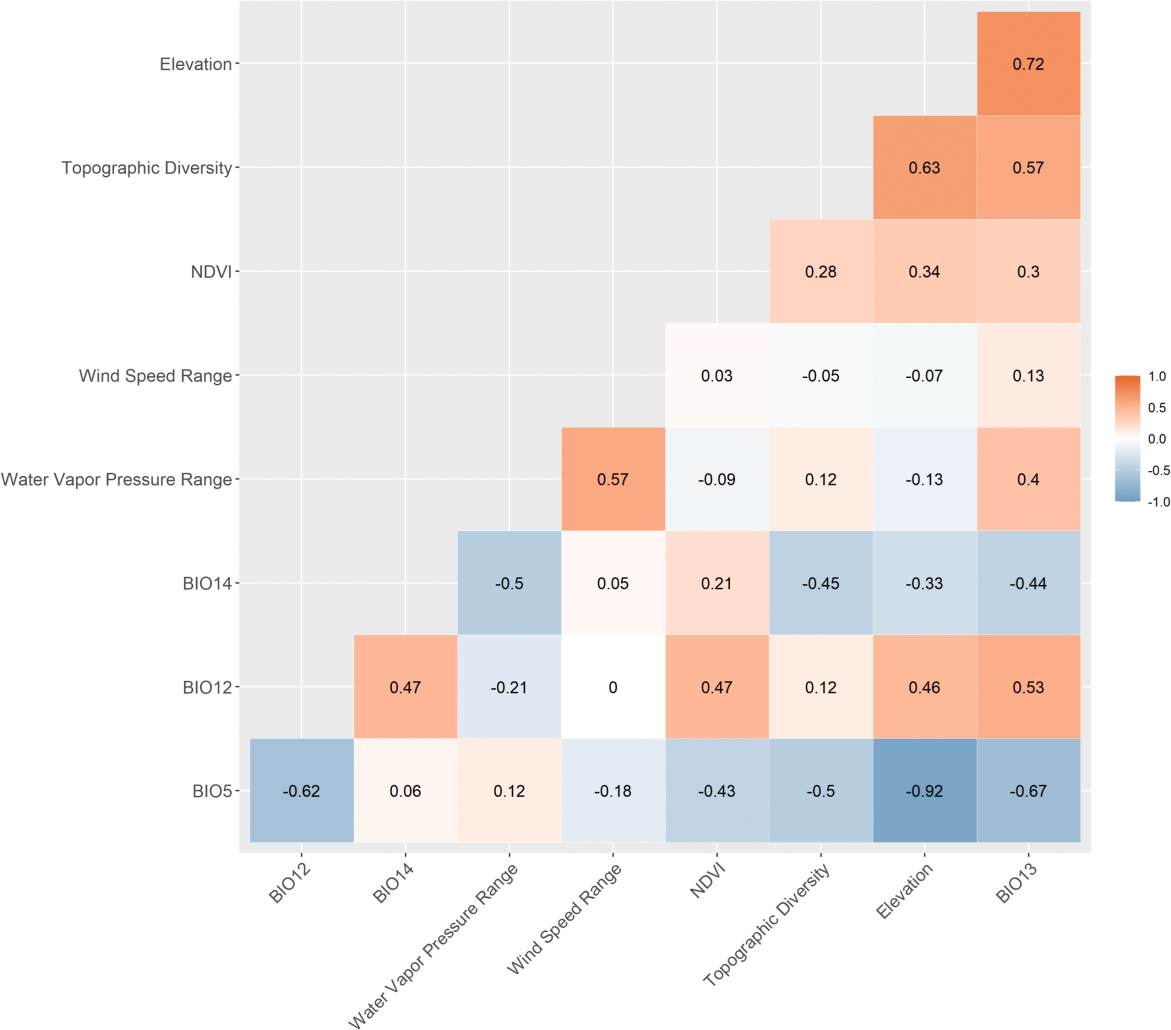
Spearman’s correlation between the bioclimatic variables. Variables: BIO 5 (Max Temperature of Warmest Month), BIO 12 (Annual Precipitation), BIO 13 (Precipitation of Wettest Month), BIO 14 (Precipitation of Driest Month), Wind Speed Range (max - min), Water Vapor Pressure Range (max - min) and Topographic diversity. Spearman correlation values -0.7 < ρ < +0.7 were considered adequate for modeling. Software: RStudio, under R programming language version 4.1.2.

After excluding the variables with the highest Spearman correlation values, retaining only those within the range of −0.7 < ρ < +0.7, the following variables were used in the modeling: BIO5, BIO12, BIO13, BIO14, wind speed range (max - min), water vapor pressure range (max - min), NDVI, and topographic diversity (Table 2 and Fig 2). Elevation was excluded from the species distribution models due to strong correlations with BIO13 (0.72) and BIO5 (-0.92). However, although this variable was not included in the modeling process, it was used for comparative analyses during model interpretation.

**Table 2:** Environmental covariables chosen in species distribution modeling. Variables Max Temperature of Warmest Month (BIO5), Annual Precipitation (BIO12), Precipitation of Wettest Month (BIO13), Precipitation of Driest Month (BIO14), Wind Speed Range (max - min) (m/s), Water Vapor Pressure Range (max - min) (kPa), Normalized Difference Vegetation Index (NDVI), and Topographic Diversity were selected based on Spearman’s correlation (–0.7 < ρ < +0.7).

Climate Variables	Landscape Variables
Max Temperature of Warmest Month (BIO 5)	Normalized Difference Vegetation Index (NDVI)
Annual Precipitation (BIO 12)	Topographic Diversity
Precipitation of Wettest Month (BIO 13)	**-----**
Precipitation of Driest Month (BIO 14)	**-----**
Wind Speed Range (max - min) (m/s)	**-----**
Water Vapor Pressure (max - min) (kPa)	**-----**

### Climatic and landscape characterization of *T. vitticeps* occurrences and their *T. cruzi* infection

The distribution of *T. vitticeps* and its *T. cruzi* infection occurrences was concentrated in the Central mesoregion, primarily in household environments. The database exhibited temporal variation in vector records and infection prevalence from 2010 to 2020, with peak captures occurring between 2013 and 2015, followed by a gradual decline after 2016 (S1 Fig). Interestingly, the prevalence of *T. cruzi* infection among triatomines exceeded the proportion of uninfected individuals (S1 Fig). The dataset counts, after applying the geographic and spatial filters, were adjusted as shown in Table 3.

**Table 3:** Environmental characterization of the occurrence points used for modeling. Environmental characterization indicates that the occurrence points are located in areas with higher temperatures (average > 18°C) and in humid to super-humid regions. The species occurs predominantly in hot, humid, and super-humid environments. Source of the Brazilian Climate Map (1:5,000,000) from IBGE (2002) [55]: https://www.ibge.gov.br/geociencias/cartas-e-mapas/informacoes-ambientais/15817-clima.html.

Database	Total	Hot (>18°C)	Sub-hot (15–18°C)	Mesothermal (10–15°C)	NDVI	Topographic Diversity
		Subdrought and Super-humid/ Humid areas				
*Triatoma* *vitticeps*	298	4/133	1/117	0/43	$\bar{x}$ = 0.7; max = 0.85; min = 0.27	$\bar{x}$ = 0.63; max = 1.0; min = 0.05
*Triatoma* *vitticeps* infected by *Trypanosoma cruzi*	237	0/86	1/114	0/36	$\bar{x}$ = 0.7; max = 0.85; min = 0.27	$\bar{x}$ = 0.62; max = 1.0; min = 0.05
TcII	28	0/17	0/10	0/1	$\bar{x}$ = 0.71; max = 0.80; min = 0.47	$\bar{x}$ = 0.68; max = 0.92; min = 0.33
TcIII	5	0/1	0/3	0/1	$\bar{x}$ = 0.74; max = 0.80; min = 0.64	$\bar{x}$ = 0.71; max = 0.88; min = 0.48
TcIV	32	0/10	0/21	0/1	$\bar{x}$ = 0.73; max = 0.81; min = 0.58	$\bar{x}$ = 0.65; max = 0.95; min = 0.28
Z3	14	0/7	0/6	0/1	$\bar{x}$ = 0.72; max = 0.78; min = 0.58	$\bar{x}$ = 0.72; max = 0.92; min = 0.33

These occurrences were concentrated mainly in the Central and South mesoregions, which have the highest mean altitudes in the state (S2 Table). Considering the landscape characterization performed for the mesoregions of the state of Espírito Santo (S3 Table), aimed at understanding land use and land cover patterns in the state [60], the results indicate that the Central and South mesoregions contain the largest forested areas. The North Coast and Northwest have the smallest, with the latter dominated by pastures and land-use mosaics, totaling 57.53% and 77.62% of their territories, respectively (S3 Table). Occurrences of *T. vitticeps* and its infections by *T. cruzi* (TcII, TcIII, TcIV and Z3) were recorded in areas with dense, healthy vegetation (NDVI with $\bar{x}$ ≥ 0.7), average altitude above 394.64 m (except for TcIII, which has an average altitude of its occurrences of 224.80 m) (S4 Table).

**Fig 3:**
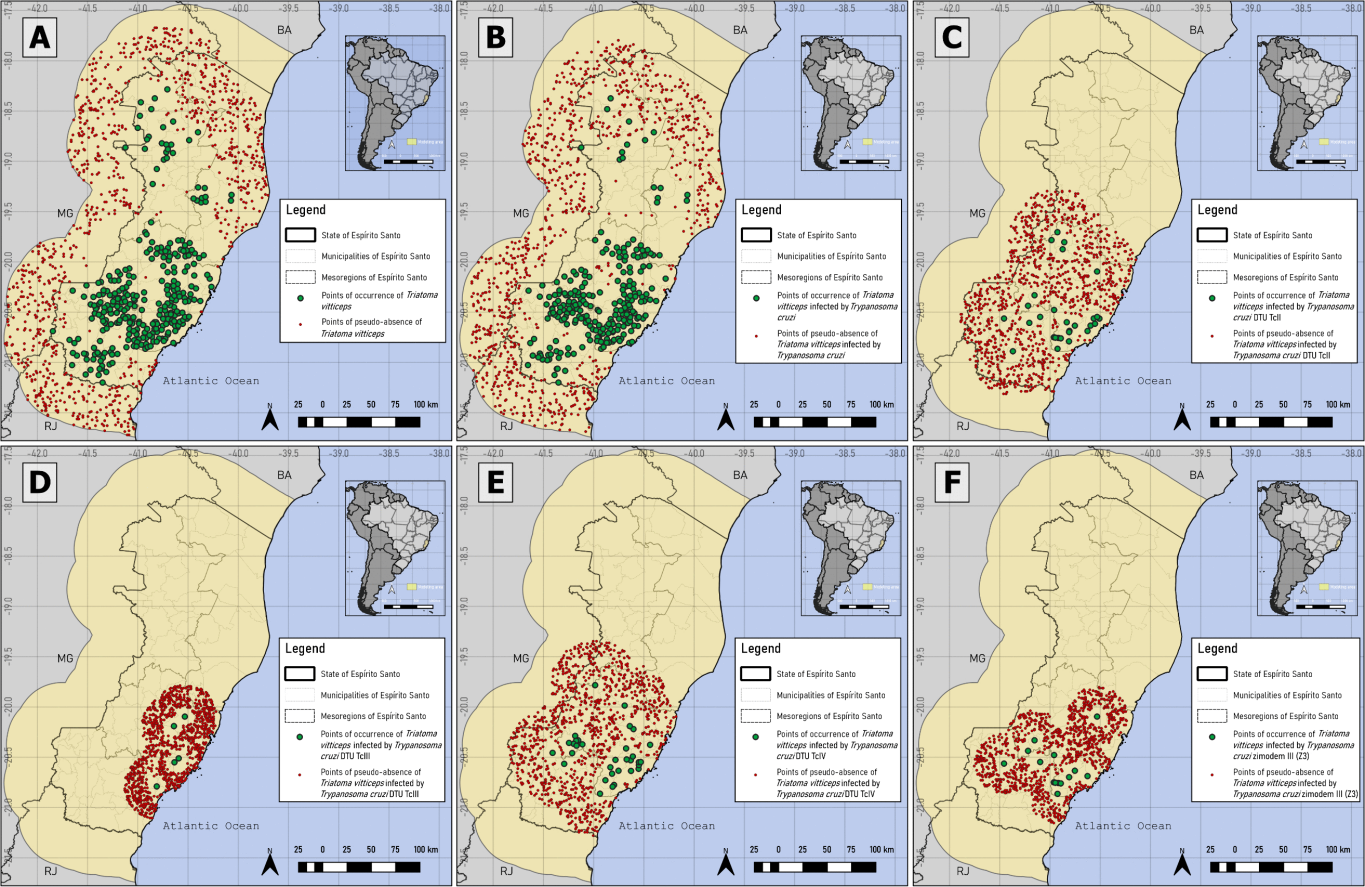
Presence and pseudo-absence for *Triatoma vitticeps*, *Trypanosoma cruzi*-like, TcII, TcIII, TcIV and Zymodeme 3. A: Points of occurrence and pseudo-absence of *T. vitticeps*; B: *T. vitticeps* infected by *Trypanosoma cruzi*-like; C: DTU TcII; D: DTU TcIII; E: DTU TcIV; and F: Zymodeme 3 (Z3). Mixed infections involving TcII and TcIII/TcIV could not be distinguished, as the PCR-RFLP assay used does not allow discrimination between these two DTUs. Software: QGIS 3.22. Source: GADM data version is 4.1. Evaluable from: https://gadm.org/download_country.html.

**Fig 4:**
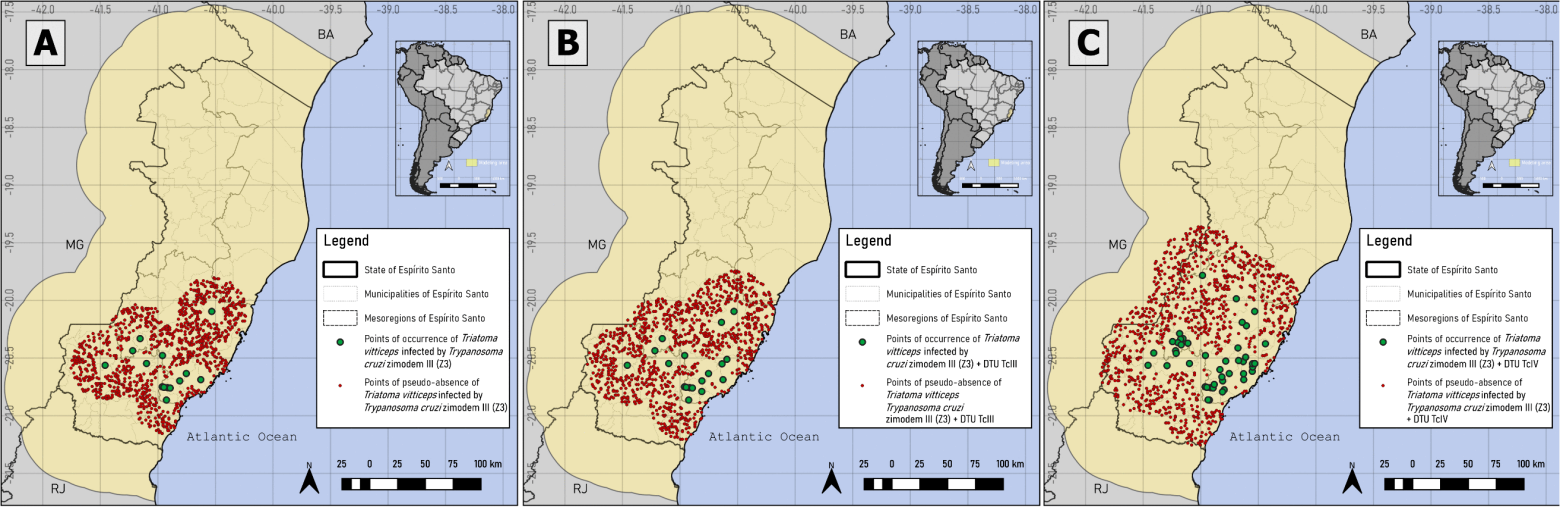
Presence and pseudo-absence for *T. vitticeps* infected by the Zymodeme 3 (Z3), Z3/TcIII and Z3/TcIV. Z3 genotype (A) and performing the combinations of the genotypes Z3 with TcIII (Z3/TcIII) (B) and Z3 with TcIV (Z3/TcIV) (C) databases. Mixed infections involving TcII and TcIII/TcIV could not be distinguished, as the PCR-RFLP assay used does not allow discrimination between these two DTUs. Software: QGIS 3.22. Source: GADM data version is 4.1. Evaluable from: https://gadm.org/download_country.html.

Pseudo-absences for *T. vitticeps* and *T. cruzi* infection were generated in areas ≤2.31% and ≤2.35%, respectively, totaling 1000 points. For TcII, TcIII, TcIV, Z3, combination of Z3 with TcIII and Z3 with TcIV occurrences, pseudo-absences were generated in suitability below 53.7%, 36.36%, 55.2%, 57%, 55.28% and 59.76%, respectively. The distribution of occurrence and pseudo-absence points for each database is shown in Figs 3 and 4.

### Modeling algorithms performance evaluation

Across all models, the best-performing algorithms were SVM, Random Forest, Maxent, and Boosted Regression Trees, whereas Mahalanobis Distance, GLM, and Domain showed the lowest performance (S5 Table). For the TcIII, TcIV, Z3, Z3–TcIII, and Z3–TcIV modeling approaches, all algorithms exhibited the largest interquartile ranges, likely due to the limited number of occurrence records available for these groups (S5 Table). GLM and Domain algorithms were not used to generate the *T. vitticeps* ensemble model because they exhibited the largest interquartile dispersions compared with the other algorithms. In addition, across all modeling approaches, partitions generated by the Mahalanobis Distance were excluded from the ensemble models due to low TSS values (< 0.7).

### Species distribution models’ characterization: *Triatoma vitticeps*, *Trypanosoma cruzi*-like and its DTUs

**Fig 5:**
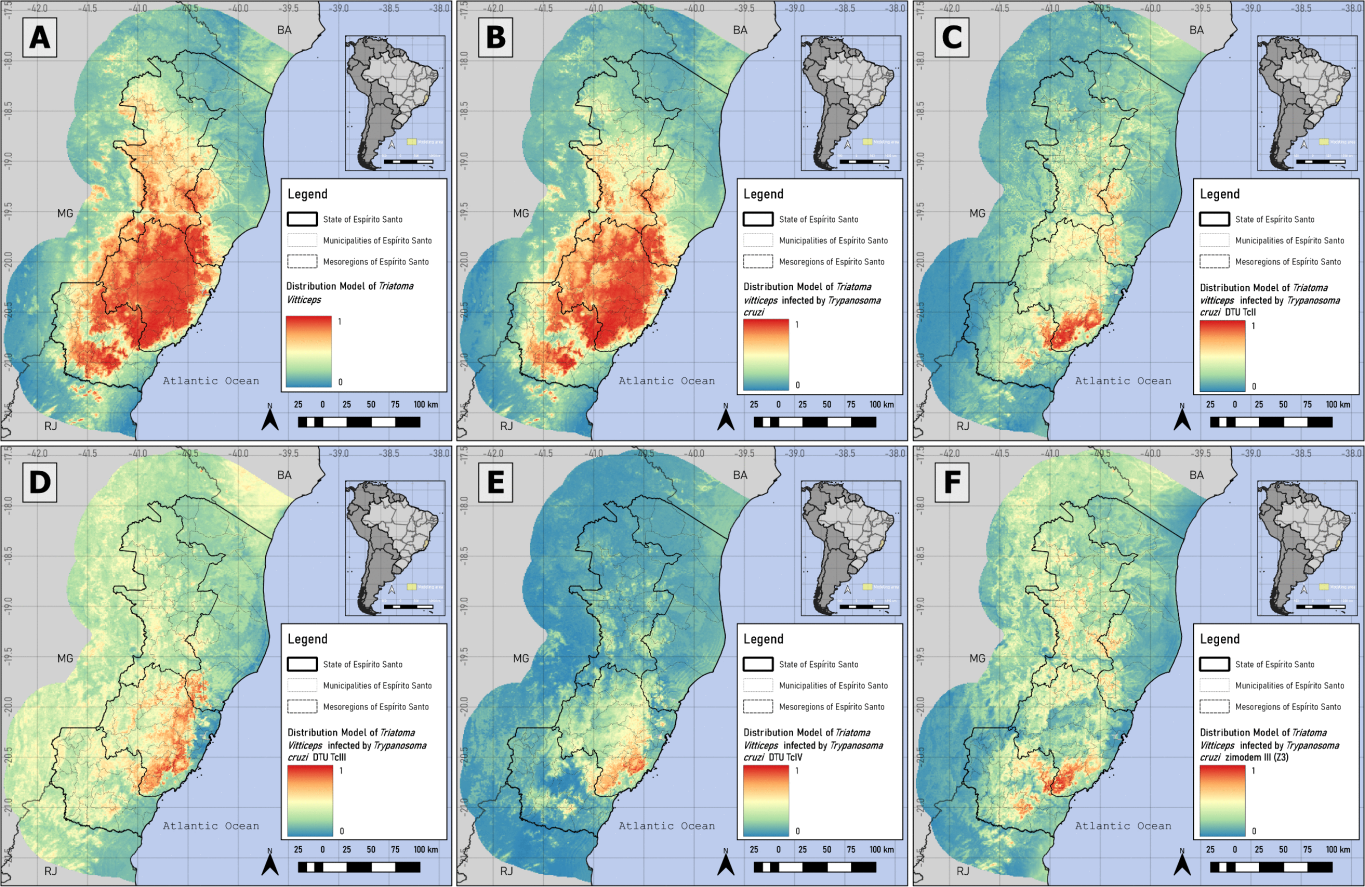
Species Distribution Modeling of *Triatoma vitticeps*, *Trypanosoma cruzi*-like, TcIII, TcIV and Zymodeme 3 (Z3). A: Species Distribution Model of *T. vitticeps*; B: *T. vitticeps* infected by *Trypanosoma cruzi*-like; C: DTU TcII; D: DTU TcIII; E: DTU TcIV; and F: Zymodeme 3. Environmental suitability ranges from 0% (blue) to 100% (red), with greater suitability in the Central mesoregion. Software: QGIS 3.22. Source: GADM data version is 4.1. Evaluable from: https://gadm.org/download_country.html.

**Fig 6:**
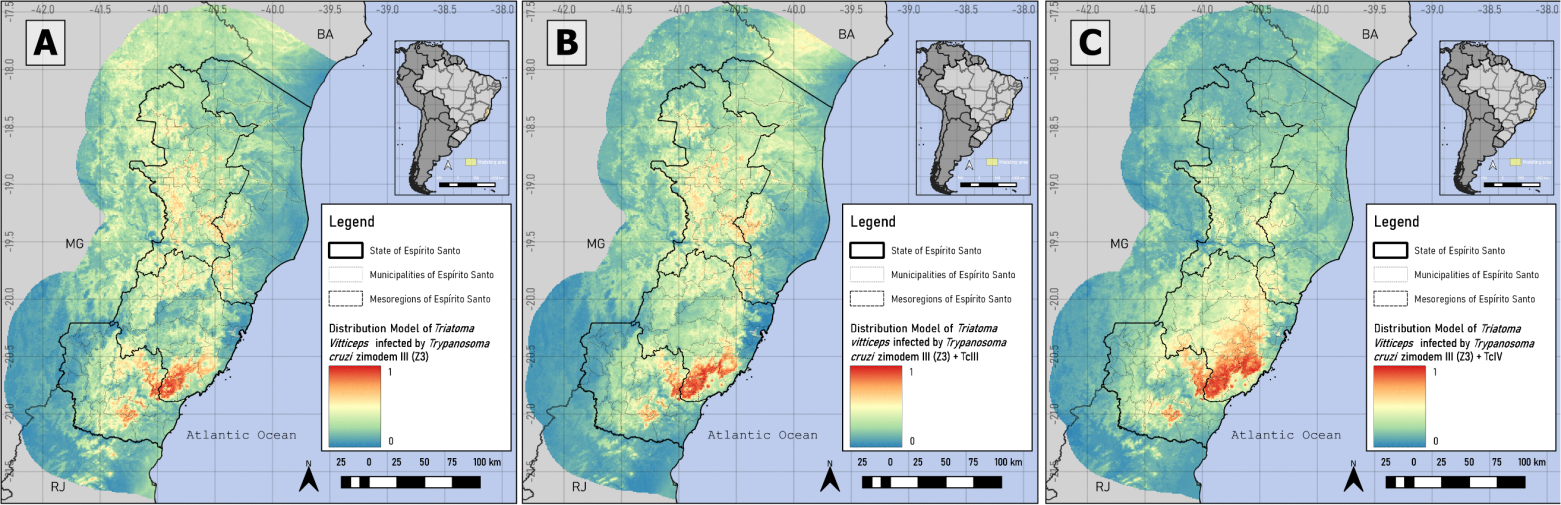
Species Distribution Modeling of *T. vitticeps* infected by Zymodeme 3 (Z3), Z3/TcIII and Z3/TcIV. Z3 genotype (A) and performing the combinations of the Z3 with TcIII (Z3/TcIII) (B) and Z3 with TcIV (Z3/TcIV) (C) databases. Environmental suitability ranges from 0% (blue) to 100% (red), with greater suitability in the Central mesoregion. Software: QGIS 3.22. Source: GADM data version is 4.1. Evaluable from: https://gadm.org/download_country.html.

As expected, the models of *T. vitticeps* and *T. cruzi-like* infection covered areas of high model suitability from all individual DTUs, with means values ($\bar{x}$) in the Central and South mesoregion ranging from 56–79% and 52–79%, respectively (S6 Table and Figs 5 and 6). The SDM for DTU TcII was concentrated in the Central-South mesoregions of Espírito Santo (Fig 5C and S6 Table), while the DTU TcIII (Fig 5D) showed a more sparse geographical projection, with the highest suitability primarily in the Central and South mesoregion (Fig 5D and S6 Table).

In contrast, the DTU TcIV model displayed a narrower distribution, extending from the Central to the South mesoregions, with lower coverage and suitability in the Northwest and North Coastal mesoregions (Fig 5E and S6 Table). The Z3 model exhibited a similar spatial pattern to TcIII and TcIV, depending on the mesoregion, also showing reduced suitability in the Northwest and North Coast (S6 Table and Figs 5E and 6A).

The SDM combining Z3 and TcIII revealed overlapping and geometrically similar areas of environmental suitability to the Z3 model, mainly in the Central and Northwest mesoregions (S6 Table and Fig 6A and 6B), whereas the model combining Z3 and TcIV indicated greater suitability in the Central mesoregion (S6 Table and Fig 6A and 6C).

The models of *T. vitticeps* and its infection by *T. cruzi* showed the highest similarity indices, mainly in the Central (0.96) and South (0.95) mesoregions, decreasing in the Northwest mesoregion (0.76) (S7C, S7D and S7A Table). DTU TcII presented the lowest similarity indices in the Northwest (0.0 – 0.52) and North Coast (0.02 – 0.58) mesoregions (S7A and S7B Table), especially when compared with TcIV. In the Central mesoregion, TcII showed the second-highest similarity with TcIV (0.62), and in the South, with Z3 (S7C and S7D Table).

In the Northwest and North Coast mesoregions TcIII displayed its highest similarity with the Z3 model, whereas TcIV had the lowest similarity with Z3 among all mesoregions (S7A–S7D Table). In contrast, in the Central mesoregion, TcIII was more similar to TcII (0.65), TcIV (0.58) and to the combined Z3 with TcIV model (0.61), while TcIV reached its highest similarity (0.75) with Z3 combined with TcIV (S7C Table). In the South, TcIII showed its lowest similarity values, and TcIV, despite low similarity with *T. vitticeps* (0.09) and *T. cruzi* (0.09) models achieved higher values with Z3 combined with TcIV (0.41), TcII (0.42), and Z3 combined with TcIII (0.61) (S7D Table).

### Classification by Decision Tree: *Triatoma vitticeps* and *Trypanosoma cruzi*-like

The classification tree showed that the highest overall classification success rate (62%) was achieved by the J48 and LMT algorithms (Table 4). Considering positive and negative classes separately, the models exhibited higher sensitivity and lower specificity, with higher recall for the positive class (true positives). The LMT algorithm achieved the best recall (0.878), identifying 84% of infected triatomines, although its precision was lower (62%) due to false positives, reducing the overall accuracy (Table 5).

**Table 4:** Overall performance of algorithms in the decision classification tree. Performance of the J48, RepTree, and LMT algorithms in the analysis of *Triatoma vitticeps* and *Trypanosoma cruzi*-like occurrence, highlighting the LMT algorithm, which identified 84% of infected triatomines despite an overall accuracy of 61.2%. Software: WEKA 3.8.6.

Algorithms	Accuracy	Sensitivity	Specificity	Precision	Recall	F-score	ROC
*J48*	62.4	80.2	35.69	0.609	0.624	0.604	0.606
*Rep Tree*	62.55	79.14	37.69	0.612	0.626	0.609	0.61
*LMT*	62.59	80.2	35.69	0.612	0.626	0.581	0.594

**Table 5:** Classification decision tree of *Triatoma vitticeps* infected and not infected by *Trypanosoma cruzi*-like. Classification of the vector as infected or uninfected based on the relationship between true positives and true negatives (TP/TN) and false positives and false negatives (FP/FN).

Algorithms	TP/ TN	FP/FN	Precision	Recall	F-score	ROC	Class
*J48*	0.357	0.198	0.546	0.357	0.432	0.606	Negative
	0.802	0.643	0.652	0.802	0.719	0.606	Positive
*REPTree*	0.377	0.209	0.546	0.377	0.446	0.61	Negative
	0.791	0.623	0.656	0.791	0.717	0.61	Positive
*LMT*	0.247	0.122	0.575	0.247	0.346	0.594	Negative
	0.878	0.753	0.636	0.878	0.738	0.594	Positive

**Fig 7:**
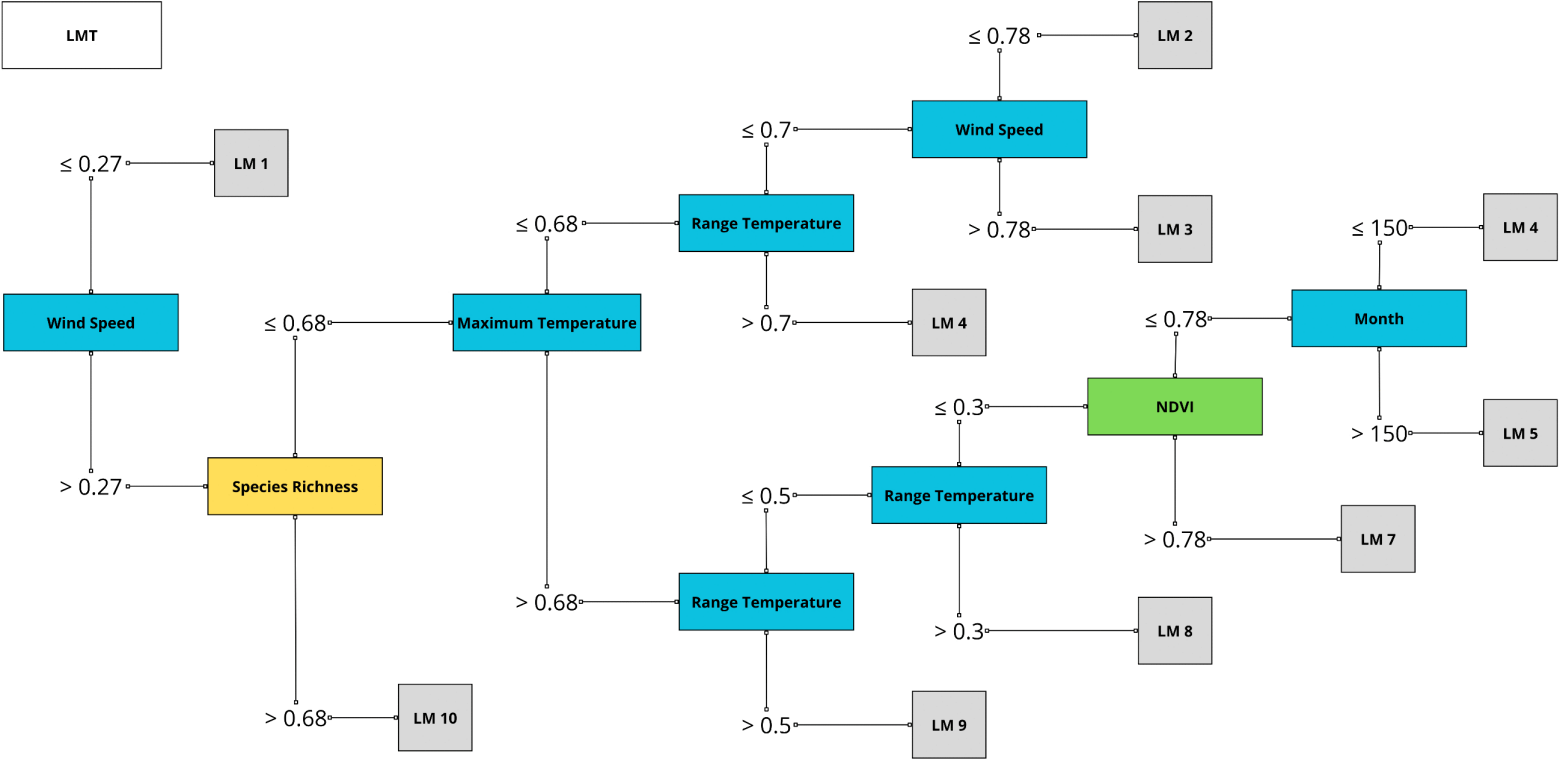
Classification model based on Logistic Model Tree (LMT) algorithm decision tree. Classification performed for *Triatoma vitticeps* infected with *Trypanosoma cruzi*-like based on the Logistic Model Tree (LMT) decision tree algorithm, with variables: wind speed, maximum temperature, range temperature, month in blue, NDVI in green and species richness in yellow. The values on the lines between the nodes represent the range of values used to classify individuals as infected or not with *T. cruzi*. Decision tree generated with Weka 3.8.6 software.

The variables that influenced the divisions at the nodes of these trees were wind speed range (max - min) and temperature (Fig 7), effectively distinguished between different groupings. The normalized values ranged from 0 and 1, with splits occurring at wind speed> or < 0.27, mammal species richness> or < 0.81, maximum temperature> or < 0.68, and minimum temperature> or < 0.81. The structure of the LMT algorithm decision tree is shown in Fig 7.

## Discussion

The maintenance and dispersion of rarely isolated DTUs remain unresolved puzzle [5]. Actually, how can we explain the maintenance in nature of *T. cruzi* genotypes that are so rarely found? What is the transmission strategy that guarantees their perpetuation? It is worth remembering that these genotypes are frequently found infecting animals that are very distant geographically, a distance that is impossible for an insect vector to cover [5].

In Brazil, among the 68 known species of triatomines, 13 are of epidemiological relevance due to their behavioral traits, including *T. vitticeps* [69]. In the state of Espírito Santo (ES), house invasion by infected triatomines is mainly caused by *T. vitticeps* in rural municipalities [25], particularly in mountainous regions with irregular terrain [34]. Although *T. vitticeps* is considered a secondary vector of *T. cruzi* due to the long interval between feeding and defecation [25], oral transmission remains possible given its high infection rates [10,11].

The occurrences of triatomine species in Espírito Santo reveal the absolute predominance of *T. vitticeps* [70], highlighting its capacity to harbor and potentially transmit multiple *T. cruzi* genotypes (TcI, TcII, TcIII and TcIV) [10,35]. This species shows the ability to migrate among three different habitats - domestic, peridomestic, and sylvatic - without evidence of population structuring [71]. The origin of the high infection rates remains unclear, as no colonies, nymphs, or eggs were found in surrounding areas [10,25,41]. The highest number of *T. vitticeps* infected with *T. cruzi* recorded in the database (S1 Table and S1 Fig) highlights its consistently high infection rates, and the peaks in captures between 2013 and 2015 likely reflect either differences in sampling effort between LABTRIP and Nemes/SESA field studies or natural population fluctuations. Both these peaks and the low capture numbers in 2020 are consistent with these explanations.

Spearman’s correlation analysis identified one temperature variable (BIO 5) and three precipitation variables (BIO 12, BIO 13 and BIO 14) within the acceptable correlation range (-0.7 ≤ ρ ≤ 0.7) (Fig 2), representing the main climatic gradients of Espírito Santo. These variables, together with wind speed range and water vapor pressure range (Table 2), are known to influence the foraging behavior, dispersal capacity, and dehydration risk of triatomine vectors under conditions of high temperature and low humidity. Collectively, they play a critical role in shaping the distribution of *T. vitticeps*, affecting its survival, mobility, and infection potential in natural environments [20,34,72–81]. Although Elevation was not directly included in the species distribution models, its influence was indirectly represented through highly correlated variables, BIO13 (0.72) and BIO5 (-0.92), allowing the models to reflect altitudinal patterns in the study area (Fig 2).

Knowing that elevation does not provide significant gains in predictive performance in species distribution models (SDMs) [82,83], it was incorporated as a comparative variable to explore altitudinal distribution patterns considering the pronounced topographic heterogeneity of Espírito Santo. Species distributions do not respond directly to elevation per se, but rather to gradients in abiotic conditions that are regulated by it [82,83]. Nonetheless, elevation can act as a useful proxy for non-climatic factors that may constrain species’ geographic distributions [84]. Given that the hypothesized displacement of triatomines in the study area is associated with higher-altitude zones of the state’s mountainous region [54], elevation represents an important variable for comparison with ecological niche models, adding ecological interpretability without compromising model performance and providing a more comprehensive environmental context for the region.

NDVI is a classical indicator of vegetation health and density derived from chlorophyll spectral reflectance [85], being an indication of anthropogenic disturbance - a relevant factor when interpreting triatomine foraging behavior, since deforestation drives these insects to seek alternative food sources due to altered local biodiversity [26,34,86]. Topographic diversity further reflects ecosystem heterogeneity and the variety of topoclimatic niches that support higher biodiversity and species persistence under climate change [87,88].

The concentration of *T. vitticeps* and its infections (S4 Table) and the high suitability indicated by the distribution models (S6 Table and S5A and S5B Fig) in the Central and South mesoregions suggests that vector movement may originate from areas with preserved vegetation (high NDVI values) and higher-altitude regions [41] characterized by greater topographic diversity (S2 and S4 Tables). This scenario reflects the prevailing land-use and land-cover patterns in these areas, which are predominantly covered by forest and pasture [60]. In contrast, the North Coast and Northwest mesoregions are characterized by agricultural mosaics and pasturelands [60], reinforcing the association between vector occurrence, infection, vegetation density, and altitude.

The North Coast and Northwest, characterized by lower altitudes (S2 Table) and the main agricultural areas of Espírito Santo [89], have undergone intense landscape fragmentation and deforestation, which affect the dynamics of mammal hosts and triatomine populations by restricting them to remnant forest patches [26,90]. This pattern supports previous evidence of an inverse relationship between mammal richness and the likelihood of *T. vitticeps* occurrence in households [41], with deforestation likely increasing home invasions by this species [70].

The observed overlap between the *T. vitticeps* distribution model and areas of *T. cruzi* infection, despite a 9% difference in environmental suitability (S6 Table), underscores a critical scenario for *T. cruzi* maintenance, given the vector’s ability to harbor five genotypes [10]. The identification of both occurrences in areas with warm and highly humid climatic conditions (Table 3) [55] may be related to triatomine blood-feeding behavior [20,81], as high humidity favors oral route over contaminative transmission, with bats acting as secondary reservoirs due to their high infection rates and insectivorous feeding habits [11].

The overlapping patterns between the model of DTU TcII and the topographic diversity (S6 Table and Fig 5C) indicates a possible prevalence in the mountainous regions of Espírito Santo, as these areas enhance species’ adaptation and resilience to climate change [88,91]. Caution is warranted when interpreting the TcII distribution model, as it identifies areas of likely presence - particularly within the Central mesoregion - that reflect the microenvironmental conditions of sampled triatomines, rather than their true origin or full ecological range, since infections may have been acquired elsewhere (Table 3 and Fig 5C and S2, S4 and S6 Tables). The wide dispersion of the TcII boxplot (S4C Table) and the high model accuracy suggest that Espírito Santo represents only a portion of the genotype’s potential distribution. Expanding occurrence data beyond the state could improve distribution delineation.

TcIII and TcIV were distinguished from the former Z3 zymodeme using PCR and sequencing [92,93]. The electrophoretic separation of *T. cruzi* subpopulations using isofunctional enzymes enabled the unequivocal discrimination of these polar subpopulations and represented a fundamental step in understanding the heterogeneity and ecology of *T. cruzi* transmission.

The TcIII and TcIV models exhibited a concentration within the Central mesoregion, encompassing diverse climatic and landscape conditions (Tables 3 and S4), which may indicate ecological resilience (patterns of the topographic diversity) - particularly in coastal municipalities - although less extensively than TcII (Fig 5C–5E). The jackknife method applied to the TcIII model allowed exploration of the limited environmental data from each occurrence, identifying suitable areas in the Central mesoregion. However, the small sample size complicates algorithm interpretation, exaggerating the suitability in this region, as reflected by dispersed interquartile ranges in the TcIII TSS boxplot (S4D Table). Despite a similar number of occurrences to TcII, TcIV models showed higher algorithmic agreement and lower interquartile ranges in TSS (S4E Table), suggesting Espírito Santo covers less of TcII’s environmental diversity.

As a summary, in all models of TcII, TcIII, and TcIV, areas of high environmental suitability correspond to regions with dense vegetation, high topographic diversity, forest cover, and mountainous terrain, predominantly in the Central and Southern mesoregions, while the North Coast, dominated by pasture and land-use mosaics (57% of area; S3 Table), has fragmented vegetation, and recent deforestation has been linked to increased home invasions by *T. vitticeps* [26,70]. Two scenarios may explain *T. vitticeps* and *T. cruzi* DTU distribution in the North Coast: (i) low environmental suitability compared to other mesoregions, with restricted suitability to areas near habitat fragments; or (ii) sampling gaps that exaggerate suitability in other regions. In any case, the North Coast remains an ecologically distinctive region, favoring generalist species like Didelphis and bats, which likely sustain the *T. cruzi* cycle [11,23,94,95].

The Z3 modeling presented a greater interpretive challenge because the Zymodeme technique does not distinguish TcIII from TcIV DTUs [92,93]. The Z3 model (Fig 5F), although showing areas of high environmental suitability overlap between the two DTUs, exhibited inconsistencies compared to the individual TcIII and TcIV models, with spatial shifts in the suitable area patches (Fig 5D–5F). All the models - Z3, Z3/TcIII and Z3/TcIV - identified high-suitability areas common to TcIII and TcIV surrounding mountainous regions and aligned with topographic diversity, similar to TcII (Figs 5 and 6). This indicates that even with limited TcIII data, potential presence locations can be inferred.

Similarity of TcIII distribution (S7A–S7D Table) suggests two possibilities: i) higher similarity with Z3 across all mesoregions due to Z3 prevalence and environmental characteristics in relation to TcIII (S7A–S7D Table); or ii) TcIII occurs more in the Northeast and North Coast than TcIV, while TcIV dominates Central and South (S7A–S7D Table). The first scenario appers plausible, but still indicates that the Z3 dataset appears mixed, predominantly with TcIV and TcIII in Central and South mesoregions, with potential TcIII influence in the Northwest and North Coast (S7A–S7D Table). The low similarity exhibited by TcII with TcIV in the Northwest and North Coast reflects that TcIV is concentrated in the Central and South mesoregions, while TcII has a broader geographic distribution (Fig 5C and 5E and S7 Table).

The high similarity between the distribution models of *T. vitticeps* and its infection by *T. cruzi* in the Central and South mesoregions (S7 Table) indicates a strong spatial overlap between vector occurrence and infected records in these areas, suggesting that infection events are widely distributed across the vector’s geographic range rather than being spatially clustered (Fig 5A and 5B). This pattern supports the relevance of *T. vitticeps* as a spatially consistent host of *T. cruzi* in these mesoregions, although it does not directly reflect transmission frequency or infection sources.

Decision tree analyses (Fig 7 and Tables 4 and 5) consistently identified wind speed, mammal richness, and maximum temperature as the variables for classifying infected *T. vitticeps*, highlighting the relevance of these explanatory variables in field planning. The LMT algorithm effectively identified infected triatomines, with environmental variables such as wind speed and temperature driving predictions. Although precision was affected by the presence of false positives (Fig 7 and Tables 4 and 5), the models’ high sensitivity supports early detection and continuous epidemiological surveillance, informing targeted control strategies [96,97]. Mammalian diversity and maximum temperature are important variables in vector dynamics, as ectothermic triatomines exhibit increased activity, reproduction, and feeding frequency at higher temperatures, highlighting their combined role in shaping population ecology [98].

The decision tree models reached a maximum accuracy of 62% (Fig 7 and Tables 4 and 5), reflecting the difficulty of classifying triatomines by *T. cruzi* infection, likely due to limitations in the temporal and spatial resolution of climatic and environmental data, as infected and uninfected vectors share habitats [99,100]. Possible explanations include: (i) using geographic coordinates, particularly regional centroids, which may misrepresent the actual distribution of infected and non-infected triatomines; and (ii) the need to incorporate comprehensive data on *T. cruzi* distribution and transmission among wild mammals, which would improve understanding of parasite circulation and strengthen the analysis. Decision trees offered a hierarchical representation of interactions among environmental and climatic factors, improving the interpretation of their influence on triatomine ecology and *T. cruzi* infection across different ecological contexts, and complementing the SDM models.

Using the coordinates of the centroid of the collection sites (S1 Table), together with a spatial resolution of 1 km² for the environmental variables, partially mitigated these uncertainties, allowing for the characterization of important climatic and landscape features associated with the vector and its *T. cruzi* infection in Espírito Santo (Table 3). Improving analyses requires refined variables capturing detailed landscape and climate information, precise geographic coordinates, and comprehensive data on *T. cruzi* distribution among wild mammals. Such integration would enhance understanding of parasite circulation and the enzootic cycle [23,38].

Wind has been identified as one of the factors influencing the flight displacement of triatomines, typically associated with low wind speeds and other factors such as temperature, humidity, and nutritional status [29,80,101–105]. The prevalence of winged adults and the absence of nymphs reinforce this idea, suggesting that the flight capacity of *T. vitticeps*, likely enhanced by the mountainous relief and prevailing air currents of the Central mesoregion, facilitates long-distance dispersal, supporting Dario et al.’s hypothesis [10,11,41]. Such displacement supports the concept of “Distantiae Transmission” of *T. cruzi*, previously attributed to anthropogenic movement of triatomines [106], but here possibly driven by abiotic environmental factors that enable the passive dispersal of *T. vitticeps* to new areas. Thus, wind speed appears to be a key factor determining the dispersal capacity of infected *T. vitticeps* in the Espírito Santo, representing a potential novel mechanism for the long-distance transmission of *T. cruzi*.

It is important to highlight that the database of *T. vitticeps* collections spans a temporal resolution of 10 years, from 2010 to 2020. Over this period, environmental characteristics, particularly land use and land cover variables such as NDVI, have changed. NDVI values were obtained from the Google Earth Engine for this period using the median of pixel values from satellite images covering the state, thus prioritizing the most frequent conditions during this timeframe. This approach partially addresses, but does not fully resolve, the numerous spatial changes occurring over time, making the interpretation of the database more sensitive when applied at larger spatial scales or in areas with significant landscape modifications.

Moreover, the sylvatic transmission cycle is continuously modified as the landscape changes [26,90], further necessitating caution when interpreting the models in association with the database. Therefore, species distribution models should be treated as parameters for identifying potential occurrence sites of a species, rather than as absolute truths.

This study indicates that health surveillance should focus on the Central and South mesoregions of Espírito Santo, where vegetation vigor, high altitudes, and favorable climatic conditions have the greatest influence on the dispersal of *T. vitticeps* and its *T. cruzi* infection. Variables such as humidity, temperature, and wind speed could affect vector movement and feeding, while the flight capacity of adult triatomines, enhanced by wind speeds and mountainous terrain, drives periods of peak dispersal. These results underscore the importance of tailored monitoring and prevention measures that take into account the unique ecological, climatic, and landscape characteristics of each region, thereby helping to reduce the risk of *T. cruzi* transmission more effectively.

Although relevant environmental variables for this distribution and infection were identified, the main finding was the spatial coverage of suitability for both the vector and its infection across the state at a local scale of 1 km², rather than being limited to the vicinity of occurrence points. Additionally, the modeling distinguished suitable areas at the DTU level within the state. These models will support fieldwork planning by indicating where collections should be conducted and identifying regions that require attention due to potential changes in the transmission cycle or proximity to anthropogenic environments. They will also contribute to solving the puzzle of sylvatic transmission in Espírito Santo, a challenge that has persisted for decades.

## Supporting information

S1 Fig Temporal occurrence of *Triatoma vitticeps* and *Trypanosoma cruzi*-like infection in Espírito Santo State (2010–2020). Bar graph showing the annual number of *T. vitticeps* specimens collected in Espírito Santo State between 2010 and 2020, categorized as infected (blue bars) or uninfected (red bars) with *T. cruzi*-like. The data reveals temporal fluctuations in both vector occurrence and infection rates, with a peak in detections between 2013 and 2015. Records without associated collection dates are grouped as “No date information”. Software: Microsoft Excel 2016.
(TIF)

S1 Table Database of *Triatoma vitticeps* occurrences and *Trypanosoma cruzi* infection status in Espírito Santo State, Brazil (2010–2020). Data were compiled from UFES, Fiocruz (LABTRIP), and Nemes/SESA records. Triatomines were identified following Lent & Wygodzinsky (1979) and examined for *T. cruzi*-like flagellates. Of 2,527 specimens, 1,530 (60.55%) were infected; 95 DTUs were identified: 36 samples corresponded to DTU TcII, 5 to DTU TcIII, 40 to DTU TcIV, and 14 to Z3. DTUs were identified using multiplex PCR of the mini-exon gene, followed by PCR-RFLP assays targeting 1f8/Alw21I (TcII) and histone 3 (H3)/AluI (TcIII and TcIV). Mixed infections involving TcII and TcIII/TcIV could not be distinguished with this method. Complementary Nemes/SESA data were included to ensure full state-wide coverage.
(XLSX)

S2 Table Mean, maximum, and minimum values of environmental variables. The variables used were BIO5, BIO12, BIO13, BIO14, Water Vapor Pressure Range, Wind Speed Range, Elevation, and Topographic Diversity by mesoregion (Central, South, North Coast, and Northwest). The Central and South regions presented the highest mean values of NDVI, elevation, topographic diversity, and wind speed range.
(XLSX)

S3 Table Landscape characterization of the Central, South, North Coast and Northwest mesoregions of the state of Espírito Santo regarding land use and cover. The Central and South mesoregions contain the largest forested areas, whereas the North Coast and Northwest have the smallest, with the latter dominated by pastures and land-use mosaics. Source: Land Use and Cover (Collection 9) of 2023 [60]. Evaluable from: https://storage.googleapis.com/mapbiomas-public/initiatives/brasil/collection_9/lclu/coverage/brasil_coverage_2023.tif.
(XLSX)

S4 Table Landscape characterization of occurrences database. Occurrences of *Triatoma vitticeps*, *T. vitticeps* infected by *Trypanosoma cruzi*-like, DTU TcII, DTU TcIII, DTU TcIV and Zymodeme 3 (Z3) through the variables Normalized Difference Vegetation Index (NDVI), Topographic Diversity and SRTM Elevation. Occurrences of *T. vitticeps* and its infections by *T. cruzi*-like were recorded in areas with dense, healthy vegetation and at higher altitudes.
(XLSX)

S5 Table Boxplot result values of the True Skill Statistics (TSS) of distribution models. Models of *Triatoma vitticeps* (A), *T. vitticeps* infected by *Trypanosoma cruzi*-like (B), DTU TcII (C), DTU TcIII (D), DTU TcIV (E), Zymodeme 3 (Z3) (F), combined *T. vitticeps* infected by Zymodeme 3 and DTU TcIII (G), and combined *T. vitticeps* infected by Zymodeme 3 and DTU TcIV (H). The algorithms with the best performance were SVM, Random Forests, Maxent and Boosted Regression Trees. Software: RStudio, in the R programming language version 4.1.2.
(XLSX)

S6 Table Mean, maximum and minimum values of environmental suitability. Values of the distribution models of *Triatoma vitticeps*, *T. vitticeps* infected by *Trypanosoma cruzi*-like, DTU TcII, DTU TcIII, DTU TcIV, Zymodeme 3, Zymodeme 3 combined with DTU TcIII and Zymodeme 3 combined with DTU TcIV by mesoregion. *T. vitticeps* occurrences and *T. cruzi*-like infections were concentrated in the Central and South mesoregions, with lower coverage in the Northwest and North Coast. DTU TcII was mainly restricted to the Central-South mesoregions, while TcIII, TcIV, and Z3 showed broader but less suitable distributions, also with lower coverage in the Northwest and North Coast.
(XLSX)

S7 Table Analysis the Schoener’s D Index and corresponding 95% confidence intervals (CI). Similarity of environmental and geographical models calculated for all pairwise combinations of *Triatoma vitticeps*, *Trypanosoma cruzi*-like, DTUs TcII, TcIII, and TcIV, Zymodeme 3 (Z3), and the combined models Z3/TcIII and Z3/TcIV across the Northwest (A), North Coast (B), Central (C), and South (D) mesoregions of Espírito Santo, Brazil. The similarity index ranges from 0.0 to 1.0, representing low to high similarity between models, respectively. The Central and South mesoregions exhibited the highest environmental and geographical similarities among genotypes. Software: Python, under version 3.13.2.
(XLSX)

S1 Appendix Google Earth Engine code to export the MOD13A2 V6 product, NDVI band, to the Atlantic Rainforest area plus 50 km. https://doi.org/10.5067/MODIS/MOD13A2.061.
(PDF)

S2 Appendix Google Earth Engine code to export the Global SRTM Topographic Diversity variable for the area of Brazil added to a 50 km buffer. https://doi.org/10.1371/journal.pone.0143619.
(PDF)

## References

[1] Guhl F, Jaramillo C, Vallejo GA, Cárdenas A-Arroyo F, Aufderheide A. Chagas disease and human migration. Mem Inst Oswaldo Cruz. 2000;95(4):553–5. doi: 10.1590/s0074-02762000000400018 10904414

[2] Ferreira LF, Jansen AM, Araújo A. Chagas disease in prehistory. An Acad Bras Cienc. 2011;83(3):1041–4. doi: 10.1590/s0001-37652011005000013 21739083

[3] Araújo A, Jansen AM, Bouchet F, Reinhard K, Ferreira LF. Parasitism, the diversity of life, and paleoparasitology. Mem Inst Oswaldo Cruz. 2003;98 Suppl 1:5–11. doi: 10.1590/s0074-02762003000900003 12687756

[4] Jansen AM, Xavier SC das C, Roque ALR. *Trypanosoma cruzi* transmission in the wild and its most important reservoir hosts in Brazil. Parasit Vectors. 2018;11(1):502. 30189896 10.1186/s13071-018-3067-2 PMC6127949

[5] Jansen AM, Xavier SCC, Roque ALR. The multiple and complex and changeable scenarios of the *Trypanosoma cruzi* transmission cycle in the sylvatic environment. Acta Trop. 2015;151:1–15. doi: 10.1016/j.actatropica.2015.07.018 26200785

[6] Ferreira I, de LM, Silva TPT. Eliminação da transmissão da doença de Chagas pelo Triatoma infestans no Brasil: um fato histórico. Rev Soc Bras Med Trop. 2006;39(5):507–9. 17160334 10.1590/s0037-86822006000500018

[7] Roque ALR. Importância dos animais domésticos sentinelas na identificação de áreas de risco de emergência de doença de Chagas. Rev Soc Bras Med Trop. 2008;41:4.

[8] Bastos CJC, Aras R, Mota G, Reis F, Dias JP, de Jesus RS, et al. Clinical outcomes of thirteen patients with acute chagas disease acquired through oral transmission from two urban outbreaks in northeastern Brazil. PLoS Negl Trop Dis. 2010;4(6):e711. doi: 10.1371/journal.pntd.0000711 20559542 PMC2886048

[9] Steindel M, Kramer Pacheco L, Scholl D, Soares M, de Moraes MH, Eger I, et al. Characterization of Trypanosoma cruzi isolated from humans, vectors, and animal reservoirs following an outbreak of acute human Chagas disease in Santa Catarina State, Brazil. Diagn Microbiol Infect Dis. 2008;60(1):25–32. doi: 10.1016/j.diagmicrobio.2007.07.016 17889480

[10] Dario MA, Rodrigues MS, Barros JH da S, Xavier SC das C, D’Andrea PS, Roque ALR, et al. Ecological scenario and *Trypanosoma cruzi* DTU characterization of a fatal acute Chagas disease case transmitted orally (Espírito Santo state, Brazil). Parasit Vectors. 2016;9(1):477. 27580853 10.1186/s13071-016-1754-4 PMC5006519

[11] Dario MA, Lisboa CV, Costa LM, Moratelli R, Nascimento MP, Costa LP, et al. High Trypanosoma spp. diversity is maintained by bats and triatomines in Espírito Santo state, Brazil. PLoS One. 2017;12(11):e0188412. doi: 10.1371/journal.pone.0188412 29176770 PMC5703495

[12] Carlos Pinto Dias J, Novaes Ramos A, Dias Gontijo E, Luquetti A, Aparecida Shikanai-Yasuda M, Rodrigues Coura J, et al. II Consenso Brasileiro em Doença de Chagas, 2015. Epidemiol E Serviços Saúde. 2016;25(21):1–10. 10.5123/S1679-49742016000500002 27869914

[13] Rocha FL, Roque ALR, de Lima JS, Cheida CC, Lemos FG, de Azevedo FC, et al. *Trypanosoma cruzi* infection in neotropical wild carnivores (Mammalia: Carnivora): at the top of the *T. cruzi* transmission chain. PLoS One. 2013;8(7):e67463. doi: 10.1371/journal.pone.0067463 23861767 PMC3701642

[14] Galvão C. Known vectors in Brazil. In: Galvão C, editor. Vectors of chagas disease in Brazil. Curitiba: Brazilian Society of Zoology. 2014. 289 p.

[15] Gurgel-Gonçalves R, Galvão C, Costa J, Peterson AT. Geographic distribution of Chagas disease vectors in Brazil based on ecological niche modeling. J Trop Med. 2012;2012:1–15. 10.1155/2012/705326 PMC3317230 22523500

[16] Noireau F, Diosque P, Jansen AM. *Trypanosoma cruzi*: adaptation to its vectors and its hosts. Vet Res. 2009;40(2):26. doi: 10.1051/vetres/2009009 19250627 PMC2695024

[17] Galvão C, Carcavallo R, Rocha DDS, Jurberg J. A checklist of the current valid species of the subfamily Triatominae Jeannel, 1919 (Hemiptera, Reduviidae) and their geographical distribution, with nomenclatural and taxonomic notes. Zootaxa. 2003;202(1):1. doi: 10.11646/zootaxa.202.1.1

[18] Lent H, Wygodzinsky PW. Revision of the Triatominae (Hemiptera, Reduviidae), and their significance as vectors of Chagas’ disease. Bull Am Mus Nat Hist. 1979;163(3). Available from: http://hdl.handle.net/2246/1282

[19] Souza R de CM de, Soares AC, Alves CL, Lorosa ES, Pereira MH, Diotaiuti L. Feeding behavior of *Triatoma vitticeps* (Reduviidae: Triatominae) in the state of Minas Gerais, Brazil. Mem Inst Oswaldo Cruz. 2011;106(1):16–22. doi: 10.1590/s0074-02762011000100003 21340350

[20] Jurberg J, Galvão C, Weirauch C, Moreira FFF. Hematophagous bugs (Reduviidae, Triatominae). In: Panizzi AR, Grazia J, editors. True bugs (Heteroptera) of the Neotropics. Dordrecht: Springer Netherlands; 2015. p. 353–93. doi: 10.1007/978-94-017-9861-7_13

[21] Carcavallo RU, Rodríguez MEF, Salvatella R, Curto de Casas SI, Sherlock I, Galvão C, et al. Habitats and related fauna. In: Carcavallo RU, Galíndez Girón I, Jurberg J, Lent H, editors. Atlas of Chagas disease vectors in the Americas, vol. II. Rio de Janeiro: Editora FIOCRUZ; 1998. p. 561–600.

[22] Richer W, Kengne P, Cortez MR, Perrineau MM, Cohuet A, Fontenille D, et al. Active dispersal by wild Triatoma infestans in the Bolivian Andes. Trop Med Int Health. 2007;12(6):759–64. doi: 10.1111/j.1365-3156.2007.01846.x 17550473

[23] Testai R, Ferreira de Siqueira M, Rocha DSB, Roque ALR, Jansen AM, Xavier SC das C. Space-environment relationship in the identification of potential areas of expansion of *Trypanosoma cruzi* infection in Didelphis aurita in the Atlantic Rainforest. PLoS One. 2023;18(7):e0288595. doi: 10.1371/journal.pone.0288595 37506103 PMC10381050

[24] Soares SBP, Leite GR, Sanches Corrêa-do-Nascimento G, Bertazo del Carro K, Fux B. Spatial modeling and risk assessment of chagas disease vector distribution in Espírito Santo, Brazil: a comprehensive approach for targeted control. Spat Spatio-Temporal Epidemiol. 2025;52:100710. 10.1016/j.sste.2025.100710 39955127

[25] Santos CB dos, Ferreira AL, Leite GR, Ferreira GEM, Rodrigues AAF, Falqueto A. Peridomiciliary colonies of *Triatoma vitticeps* (Stal, 1859) (Hemiptera, Reduviidae, Triatominae) infected with *Trypanosoma cruzi* in rural areas of the state of Espírito Santo, Brazil. Mem Inst Oswaldo Cruz. 2005;100(5):471–3. 16184222 10.1590/s0074-02762005000500004

[26] Cardozo M, Fiad FG, Crocco LB, Gorla DE. Effect of habitat fragmentation on rural house invasion by sylvatic triatomines: a multiple landscape-scale approach. PLoS Negl Trop Dis. 2021;15(7):e0009579. doi: 10.1371/journal.pntd.0009579 34260588 PMC8312942

[27] Moura M da S, Silva LB da, Madeira FP, Neves FWO das, Menezes ALR, Rosa JA da, et al. Flying to the moon: Impactful accounts of triatomines invasion from the 2nd to the 13th floor of an urban residential building in the municipality of Rio Branco, Acre, Brazil. Rev Soc Bras Med Trop. 2024;57:e00415. 10.1590/0037-8682-0122-2024 PMC11374126 39230162

[28] Moraes MH da S, Jesus AC de, Madeira FP, Moresco GG, Oliveira J de, Rosa JA da, et al. Triatominae (Hemiptera, Reduviidae) in homes: report of their occurrence in the municipality of Cruzeiro do Sul, Acre, South Western Amazon. Rev Soc Bras Med Trop. 2020;54:e20200296. doi: 10.1590/0037-8682-0296-2020 33206885 PMC7670736

[29] Gonçalves TCM, Victório VMN, Jurberg J, Cunha V. Biologia do *Triatoma vitticeps* (Stal, 1859) em condições de laboratório (Hemiptera: Reduviidae: Triatominae): II. Resistência ao jejum. Mem Inst Oswaldo Cruz. 1989;84(1):131–4. doi: 10.1590/s0074-02761989000100023 2181243

[30] Zeledón R, Alvarado R, Jirón LF. Observations on the feeding and defecation patterns of three triatomine species (Hemiptera: Reduviidae). Acta Trop. 1977;34(1):65–77. 16468

[31] López AG, Cardozo M, Oscherov EB, Crocco LB. Dynamics of feeding and defecation behavior of Triatoma infestans hybrids. Parasitol Res. 2020;119(9):2775–81. doi: 10.1007/s00436-020-06822-0 32737590

[32] Dias E. Observações sôbre eliminação de dejeções e tempo de sucção em alguns triatomíneos sul-americanos. Mem Inst Oswaldo Cruz. 1956;54(1):115–24. doi: 10.1590/s0074-02761956000100006 13369150

[33] Santos CB dos, Leite GR, Ferreira GEM, Ferreira AL. Infecção natural de *Triatoma vitticeps* (Stal, 1859) por flagelados morfologicamente semelhantes a *Trypanosoma cruzi* (Chagas, 1909) no Estado do Espírito Santo. Rev Soc Bras Med Trop. 2006;39(1):89–91. 16501776 10.1590/s0037-86822006000100019

[34] Leite GR, dos Santos CB, Falqueto A. Influence of the landscape on dispersal of sylvatic triatomines to anthropic habitats in the Atlantic Forest. J Biogeogr. 2011;38(4):651–63.

[35] Dario MA, Andrade TES, Dos Santos CB, Fux B, Brandão AA, Falqueto A. Molecular characterization of *Trypanosoma cruzi* samples derived from *Triatoma vitticeps* and Panstrongylus geniculatus of the Atlantic rainforest, southeast Brazil. Parasite. 2018;25:59. doi: 10.1051/parasite/2018060 30474600 PMC6254102

[36] Peterson AT, Pearson R, Anderson R, Martínez-Meyer E, Nakamura M, Bastos Araújo M, et al. Ecological niches and geographic distributions. Princeton (NJ): Princeton University Press; 2011. 328 p.

[37] Ferro E Silva AM, Sobral-Souza T, Vancine MH, Muylaert RL, de Abreu AP, Pelloso SM, et al. Spatial prediction of risk areas for vector transmission of *Trypanosoma cruzi* in the State of Paraná, southern Brazil. PLoS Negl Trop Dis. 2018;12(10):e0006907. doi: 10.1371/journal.pntd.0006907 30365486 PMC6221357

[38] Villalobos G, Nava-Bolaños A, De Fuentes-Vicente JA, Téllez-Rendón JL, Huerta H, Martínez-Hernández F, et al. A reduction in ecological niche for *Trypanosoma cruzi*-infected triatomine bugs. Parasit Vectors. 2019;12(1):240. doi: 10.1186/s13071-019-3489-5 31097007 PMC6524312

[39] Sandoval-Ruiz CA, Zumaquero-Rios JL, Rojas-Soto OR. Predicting geographic and ecological distributions of triatomine species in the southern Mexican state of Puebla using ecological niche modeling. J Med Entomol. 2008;45(3):540–6. doi: 10.1603/0022-2585(2008)45[540:pgaedo]2.0.co;2 18533450

[40] Costa J, Peterson AT. Ecological niche modeling as a tool for understanding distributions and interactions of vectors, hosts, and etiologic agents of Chagas disease. In: Mylonakis E, Ausubel FM, Gilmore M, Casadevall A, editors. Recent advances on model hosts. New York (NY): Springer; 2012. p. 59–70. 10.1007/978-1-4419-5638-5_7 22127886

[41] Dario MA, Maranhão PHC, Dos Santos GQ, Rocha M de M, Falqueto A, Da Silva LFCF, et al. Environmental influence on *Triatoma vitticeps* occurrence and *Trypanosoma cruzi* infection in the Atlantic Forest of south-eastern Brazil. Geospat Health. 2021;16(2):10.4081/gh.2021.997. doi: 10.4081/gh.2021.997 34726032

[42] Choi RY, Coyner AS, Kalpathy-Cramer J, Chiang MF, Campbell JP. Introduction to machine learning, neural networks, and deep learning. Transl Vis Sci Technol. 2020;9(2):14. 10.1167/tvst.9.2.14 PMC7347027 32704420

[43] Wazid M, Das AK, Chamola V, Park Y. Uniting cyber security and machine learning: advantages, challenges and future research. ICT Express. 2022;8(3):313–21. doi: 10.1016/j.icte.2022.04.007

[44] Greener JG, Kandathil SM, Moffat L, Jones DT. A guide to machine learning for biologists. Nat Rev Mol Cell Biol. 2022;23(1):40–55. doi: 10.1038/s41580-021-00407-0 34518686

[45] Lee YW, Choi JW, Shin E-H. Machine learning model for predicting malaria using clinical information. Comput Biol Med. 2021;129:104151. doi: 10.1016/j.compbiomed.2020.104151 33290932

[46] Barratt JLN, Sapp SGH. Machine learning-based analyses support the existence of species complexes for Strongyloides fuelleborni and Strongyloides stercoralis. Parasitology. 2020;147(11):1184–95. doi: 10.1017/S0031182020000979 32539880 PMC7443747

[47] Ligda P, Claerebout E, Kostopoulou D, Zdragas A, Casaert S, Robertson LJ, et al. *Cryptosporidium* and *Giardia* in surface water and drinking water: Animal sources and towards the use of a machine-learning approach as a tool for predicting contamination. Environ Pollut. 2020;264:114766. doi: 10.1016/j.envpol.2020.114766 32417583

[48] Ikerionwu C, Ugwuishiwu C, Okpala I, James I, Okoronkwo M, Nnadi C, et al. Application of machine and deep learning algorithms in optical microscopic detection of *Plasmodium*: a malaria diagnostic tool for the future. Photodiagnosis Photodyn Ther. 2022;40:103198. doi: 10.1016/j.pdpdt.2022.103198 36379305

[49] Zare M, Akbarialiabad H, Parsaei H, Asgari Q, Alinejad A, Bahreini MS, et al. A machine learning-based system for detecting leishmaniasis in microscopic images. BMC Infect Dis. 2022;22(1):48. doi: 10.1186/s12879-022-07029-7 35022031 PMC8754077

[50] Ekins S, de Siqueira-Neto JL, McCall L-I, Sarker M, Yadav M, Ponder EL, et al. Machine learning models and pathway genome data base for *Trypanosoma cruzi* drug discovery. PLoS Negl Trop Dis. 2015;9(6):e0003878. doi: 10.1371/journal.pntd.0003878 26114876 PMC4482694

[51] Glidden CK, Murran AR, Silva RA, Castellanos AA, Han BA, Mordecai EA. Phylogenetic and biogeographical traits predict unrecognized hosts of zoonotic leishmaniasis. PLoS Negl Trop Dis. 2023;17(5):e0010879. doi: 10.1371/journal.pntd.0010879 37256857 PMC10231829

[52] Phang WK, Hamid MHBA, Jelip J, Mudin RNB, Chuang T-W, Lau YL, et al. Predicting Plasmodium knowlesi transmission risk across Peninsular Malaysia using machine learning-based ecological niche modeling approaches. Front Microbiol. 2023;14:1126418. doi: 10.3389/fmicb.2023.1126418 36876062 PMC9977793

[53] Instituto Brasileiro de Geografia e Estatística (IBGE). Mapa político dos estados do Espírito Santo e Rio de Janeiro [map]. Rio de Janeiro: IBGE; 2015. Available from: https://geoftp.ibge.gov.br/cartas_e_mapas/mapas_estaduais_e_distrito_federal/politico/2015/rj_es_politico750k_2015.pdf

[54] Amarante OAC, Silva F de JL da, Andrade PEP de, Parecy E. Atlas Eólico: Espírito Santo. Vitória, ES: ASPE; 2009. 100 p.

[55] Instituto Brasileiro de Geografia e Estatística (IBGE). Mapa de clima do Brasil [mapa]. Rio de Janeiro: IBGE; 2002. Available from: https://www.ibge.gov.br/geociencias/cartas-e-mapas/informacoes-ambientais/15817-clima.html?edicao=15887&t=acesso-ao-produto

[56] Fernandes O, Santos SS, Cupolillo E, Mendonça B, Derre R, Junqueira AC, et al. A mini-exon multiplex polymerase chain reaction to distinguish the major groups of *Trypanosoma cruzi* and T. rangeli in the Brazilian Amazon. Trans R Soc Trop Med Hyg. 2001;95(1):97–9. doi: 10.1016/s0035-9203(01)90350-5 11280078

[57] Aliaga C, Brenière SF, Barnabé C. Further interest of miniexon multiplex PCR for a rapid typing of *Trypanosoma cruzi* DTU groups. Infect Genet Evol. 2011;11(5):1155–8. doi: 10.1016/j.meegid.2010.11.013 21255686

[58] Rozas M, De Doncker S, Adaui V, Coronado X, Barnabé C, Tibyarenc M, et al. Multilocus polymerase chain reaction restriction fragment--length polymorphism genotyping of *Trypanosoma cruzi* (Chagas disease): taxonomic and clinical applications. J Infect Dis. 2007;195(9):1381–8. doi: 10.1086/513440 17397011

[59] Westenberger SJ, Barnabé C, Campbell DA, Sturm NR. Two hybridization events define the population structure of *Trypanosoma cruzi*. Genetics. 2005;171(2):527–43. doi: 10.1534/genetics.104.038745 15998728 PMC1456769

[60] Projeto MapBiomas. Coleção 9 da Série Anual de Mapas de Cobertura e Uso da Terra do Brasil; 2023. Available from: https://storage.googleapis.com/mapbiomas-public/initiatives/brasil/collection_9/lclu/coverage/brasil_coverage_2023.tif

[61] Sánchez-Tapia A, Ribeiro Mortara S, Rocha Bezerra DS, Barros FSM, Fonseca EM, Silva ES, et al. modleR: a modular workflow to perform ecological niche modelling in R. bioRxiv [Preprint]. 2020. 10.1101/2020.04.01.021105

[62] Roberts DR, Bahn V, Ciuti S, Boyce MS, Elith J, Guillera‐Arroita G, et al. Cross‐validation strategies for data with temporal, spatial, hierarchical, or phylogenetic structure. Ecography. 2017;40(8):913–29. doi: 10.1111/ecog.02881

[63] Allouche O, Tsoar A, Kadmon R. Assessing the accuracy of species distribution models: prevalence, kappa and the true skill statistic (TSS). J Appl Ecol. 2006;43(6):1223–32.

[64] Barve N, Barve V, Jiménez-Valverde A, Lira-Noriega A, Maher SP, Peterson AT. The crucial role of the accessible area in ecological niche modeling and species distribution modeling. Ecol Model. 2011;222(11):1810–9.

[65] Barbet‐Massin M, Jiguet F, Albert CH, Thuiller W. Selecting pseudo‐absences for species distribution models: how, where and how many? Methods Ecol Evol. 2012;3(2):327–38. doi: 10.1111/j.2041-210x.2011.00172.x

[66] Witten IH, Frank E, Hall MA, Pal CJ. Data mining: practical machine learning tools and techniques. 4th ed. Morgan Kaufmann Publishers, Inc; 2016.

[67] Salzberg SL. C4.5: Programs for machine learning by J. Ross Quinlan. Mach Learn. 1994;16(3):235–40.

[68] Landwehr N, Hall M, Frank E. Logistic model trees. Mach Learn. 2005;59(1):161–205.

[69] Health Surveillance Secretariat. Ministry of Health. Acute Chagas disease and spatial distribution of triatomines of epidemiological importance, Brazil 2012 to 2016. Epidemiol Bull. 2017;50(2):1–10. Available from: https://www.gov.br/saude/pt-br/assuntos/saude-de-a-a-z/d/doenca-de-chagas/arquivos/boletim-epidemiologico-vol-50-no-02-2019-doenca-de-chagas-aguda-e-distribuicao-espacial-dos-triatomineos-de-importancia-epidemiologica-brasil-2012-a-2016.pdf/view

[70] Dias JCP, Feitosa VR, Ferraz Filho A do N, Rodrigues VLC, Alencar SA de, Sessa PA. Fonte alimentar e potencial vetorial de *Triatoma vitticeps* (Stal, 1859) com relação à doença de Chagas humana no estado do Espírito Santo, Brasil (Hemiptera, Reduviidae). Mem Inst Oswaldo Cruz. 1989;84(suppl 4):165–73. doi: 10.1590/s0074-02761989000800032

[71] Souza R de CM de, Barbosa SE, Sonoda IV, Azeredo BV de M, Romanha ÁJ, Diotaiuti L. Population dynamics of *Triatoma vitticeps* (Stål, 1859) in Itanhomi, Minas Gerais, Brazil. Mem Inst Oswaldo Cruz. 2008;103:14–20. 18368232 10.1590/s0074-02762008000100002

[72] Forattini OP, Ferreira OA, Silva EO da R e, Rabello EX. Aspectos ecológicos da tripanossomíase americana: XII - Variação regional da tendência de Panstrongylus megistus à domiciliação. Rev Saúde Pública. 1978;12(2):209–33. 102020

[73] Santos JED Jr, Viola MG, Lorosa ES, Machado EM de M, Ruas Neto AL, Corseuil E. Evaluation of natural foci of Panstrongylus megistus in a forest fragment in Porto Alegre, State of Rio Grande do Sul, Brazil. Rev Soc Bras Med Trop. 2013;46(5):575–83. doi: 10.1590/0037-8682-0149-2013 24270248

[74] Forattini OP, Barata JMS, Santos JLF, Silveira AC. Hábitos alimentares, infecção natural e distribuição de triatomíneos domiciliados na região central do Brasil. Rev Saúde Pública. 1982;16(4):171–204. doi: 10.1590/s0034-89101982000400001 6818674

[75] Forattini OP. Biogeografia, origem e distribuição da domiciliação de triatomíneos no Brasil. Rev Saúde Pública. 2006;40(6):964–98. doi: 10.1590/s0034-89102006000700004 17173153

[76] Forattini OP, Ferreira OA, Silva EO da R e, Rabello EX. Ecological aspects of American Trypanosomiasis: XII—Regional variation of the tendency of *Panstrongylus megistus* to domiciliation. Rev Public Health. 1978;12(2):209–33. 102020

[77] Curto de Casas SI, Carcavallo RU. Climate change and vector-borne diseases distribution. Soc Sci Med. 1995;40(11):1437–40. doi: 10.1016/0277-9536(95)00040-e 7667648

[78] Abrahan LB, Gorla DE, Catalá SS. Dispersal of Triatoma infestans and other Triatominae species in the arid Chaco of Argentina: flying, walking or passive carriage? The importance of walking females. Mem Inst Oswaldo Cruz. 2011;106(2):232–9. doi: 10.1590/s0074-02762011000200019 21537686

[79] Fimbres-Macias JP, Harris TA, Hamer SA, Hamer GL. Phenology and environmental predictors of Triatoma sanguisuga dispersal in east-central Texas, United States. Acta Trop. 2023;240:106862. doi: 10.1016/j.actatropica.2023.106862 36787862

[80] Vazquez-Prokopec GM, Ceballos LA, Marcet PL, Cecere MC, Cardinal MV, Kitron U, et al. Seasonal variations in active dispersal of natural populations of Triatoma infestans in rural north-western Argentina. Med Vet Entomol. 2006;20(3):273–9. doi: 10.1111/j.1365-2915.2006.00637.x 17044877 PMC1894892

[81] Guarneri AA, Lazzari C, Diotaiuti L, Lorenzo MG. The effect of relative humidity on the behaviour and development of Triatoma brasiliensis. Physiol Entomol. 2002;27(2):142–7.

[82] Hof AR, Jansson R, Nilsson C. The usefulness of elevation as a predictor variable in species distribution modelling. Ecol Model. 2012;246:86–90. doi: 10.1016/j.ecolmodel.2012.07.028

[83] Guillaume AS, Leempoel K, Rogivue A, Gugerli F, Parisod C, Joost S. Integrating very high resolution environmental proxies in genotype-environment association studies. Evol Appl. 2024;17(7):e13737. doi: 10.1111/eva.13737 38948540 PMC11212006

[84] Remonti L, Balestrieri A, Prigioni C. Altitudinal gradient of Eurasian otter (Lutra lutra) food niche in Mediterranean habitats. Can J Zool. 2009;87(4):285–91.

[85] Moreira M. Fundamentals of remote sensing and application methodologies. 4th ed. Viçosa: UFV; 2011.

[86] Schofield CJ, Diotaiuti L, Dujardin JP. The process of domestication in Triatominae. Mem Inst Oswaldo Cruz. 1999;94 Suppl 1:375–8. doi: 10.1590/s0074-02761999000700073 10677759

[87] Theobald DM, Harrison-Atlas D, Monahan WB, Albano CM. Ecologically-relevant maps of landforms and physiographic diversity for climate adaptation planning. PLoS One. 2015;10(12):e0143619. doi: 10.1371/journal.pone.0143619 26641818 PMC4671541

[88] Lawrence A, Hoffmann S, Beierkuhnlein C. Topographic diversity as an indicator for resilience of terrestrial protected areas against climate change. Glob Ecol Conserv. 2021;25:e01445. doi: 10.1016/j.gecco.2020.e01445

[89] Instituto Jones dos Santos Neves (IJSN). Boletim Técnico: Agricultura Capixaba. Vitória, ES: Instituto Jones dos Santos Neves (IJSN); 2014. Available from: https://ijsn.es.gov.br/publicacoes/boletins/boletim-tecnico-agricultura-capixaba

[90] Andrén H. Effects of habitat fragmentation on birds and mammals in landscapes with different proportions of suitable habitat: a review. Oikos. 1994;71(3):355–66.

[91] Loarie SR, Duffy PB, Hamilton H, Asner GP, Field CB, Ackerly DD. The velocity of climate change. Nature. 2009;462(7276):1052–5. doi: 10.1038/nature08649 20033047

[92] Brisse S, Barnabé C, Tibayrenc M. Identification of six *Trypanosoma cruzi* phylogenetic lineages by random amplified polymorphic DNA and multilocus enzyme electrophoresis. Int J Parasitol. 2000;30(1):35–44. doi: 10.1016/s0020-7519(99)00168-x 10675742

[93] Brisse S, Verhoef J, Tibayrenc M. Characterisation of large and small subunit rRNA and mini-exon genes further supports the distinction of six *Trypanosoma cruzi* lineages. Int J Parasitol. 2001;31(11):1218–26. doi: 10.1016/s0020-7519(01)00238-7 11513891

[94] Cáceres NC. Use of the space by the opossum Didelphis aurita Wied-Newied (Mammalia, Marsupialia) in a mixed forest fragment of southern Brazil. Rev Bras Zool. 2003;20:315–22.

[95] Cáceres NC, de Moraes Weber M, Melo GL, Meloro C, Sponchiado J, Carvalho RDS, et al. Which factors determine spatial segregation in the South American Opossums (Didelphis aurita and D. albiventris)? An ecological niche modelling and geometric morphometrics approach. PLoS One. 2016;11(6):e0157723. doi: 10.1371/journal.pone.0157723 27336371 PMC4919065

[96] Rabinovich JE, Alvarez Costa A, Muñoz IJ, Schilman PE, Fountain-Jones NM. Machine-learning model led design to experimentally test species thermal limits: the case of kissing bugs (Triatominae). PLoS Negl Trop Dis. 2021;15(3):e0008822. doi: 10.1371/journal.pntd.0008822 33684127 PMC7971882

[97] Abdi YH, Abdullahi YB, Abdi MS, Bashir SG, Ahmed NI. Using artificial intelligence in vector control: a new path for public health. J Vector Borne Dis. 2025:10.4103/jvbd.jvbd_144_25. doi: 10.4103/jvbd.jvbd_144_25 40938304

[98] Rabinovich JE. Morphology, life cycle, environmental factors and fitness – a machine learning analysis in kissing bugs (Hemiptera, Reduviidae, Triatominae). Front Ecol Evol. 2021;9:651683. doi: 10.3389/fevo.2021.651683

[99] Chabot-Couture G, Nigmatulina K, Eckhoff P. An environmental data set for vector-borne disease modeling and epidemiology. PLoS One. 2014;9(4):e94741. doi: 10.1371/journal.pone.0094741 24755954 PMC3995884

[100] Murillo-Solano C, López-Domínguez J, Gongora R, Rojas-Gulloso A, Usme-Ciro J, Perdomo-Balaguera E, et al. Diversity and interactions among triatomine bugs, their blood feeding sources, gut microbiota and *Trypanosoma cruzi* in the Sierra Nevada de Santa Marta in Colombia. Sci Rep. 2021;11(1):12306. doi: 10.1038/s41598-021-91783-2 34112903 PMC8192545

[101] Schmidt JO, Miller ML, Klotz SA. Seasonal flight pattern of the kissing bugs Triatoma rubida and T. protracta (Hemiptera: Reduviidae: Triatominae) in Southern Arizona, United States. Insects. 2022;13(3):265. doi: 10.3390/insects13030265 35323563 PMC8948865

[102] Di Iorio O, Gürtler RE. Seasonality and temperature-dependent flight dispersal of Triatoma infestans (Hemiptera: Reduviidae) and other vectors of chagas disease in Western Argentina. J Med Entomol. 2017;54(5):1285–92. doi: 10.1093/jme/tjx109 28605522

[103] Cecere MC, Vazquez-Prokopec GM, Gürtler RE, Kitron U. Spatio-temporal analysis of reinfestation by Triatoma infestans (Hemiptera: Reduviidae) following insecticide spraying in a rural community in northwestern Argentina. Am J Trop Med Hyg. 2004;71(6):803–10. doi: 10.4269/ajtmh.2004.71.803 15642975 PMC1351234

[104] Ekkens DB. Nocturnal flights of Triatoma (Hemiptera: Reduviidae) in Sabino Canyon, Arizona: I. Light collections. J Med Entomol. 1981;18(3):211–27.

[105] Gurevitz JM, Ceballos LA, Kitron U, Gürtler RE. Flight initiation of Triatoma infestans (Hemiptera: Reduviidae) under natural climatic conditions. J Med Entomol. 2006;43(2):143–50. doi: 10.1603/0022-2585(2006)043[0143:fiotih]2.0.co;2 16619592 PMC1894897

[106] Xavier SC das C, Roque ALR, Bilac D, de Araújo VAL, da Costa Neto SF da C, Lorosa ES, et al. Distantiae transmission of *Trypanosoma cruzi*: a new epidemiological feature of acute Chagas disease in Brazil. PLoS Negl Trop Dis. 2014;8(5):e2878. doi: 10.1371/journal.pntd.0002878 24854494 PMC4031066

