# Supplementary material for: Unveiling the ecology and spatial dynamics of Trypanosoma cruzi, its DTUs and Triatoma vitticeps in the Atlantic Forest of south-eastern Espírito Santo State, Brazil
Source: PLoS Negl Trop Dis. 2026 Mar 16;20(3):e0014111. doi: 10.1371/journal.pntd.0014111 (PMC13004508; doi:10.1371/journal.pntd.0014111)
Supplement: S1 Appendix — https://doi.org/10.5067/MODIS/MOD13A2.061. (PDF) [file pntd.0014111.s009.pdf]

```
/*=================================
     Beginning of Code
==================================== */

var dataset = ee.ImageCollection('MODIS/006/MOD13A2')
                  .filter(ee.Filter.date('2010-01-01', '2020-03-01'));

var ndvi = dataset.median().clip(ES).toFloat().select('NDVI');

var ndviVis = {
  min: -2000,
  max: 10000.0,
  palette: [
    'FFFFFF', 'CE7E45', 'DF923D', 'F1B555', 'FCD163', '99B718', '74A901',
    '66A000', '529400', '3E8601', '207401', '056201', '004C00', '023B01',
    '012E01', '011D01', '011301'
  ],
};

Map.setCenter(-42.93, -20.19, 4);
Map.addLayer(ndvi, ndviVis, 'NDVI');

var orig = dataset.first().projection();
var x = ndvi.reproject(orig)
Export.image.toDrive({
  image: ndvi,
  description: 'NDVI_ES_January2007_to_March2020',
  scale: x.projection().nominalScale().getInfo(),
  crs: 'EPSG: 4326',
  folder: 'EE',
  maxPixels: 49338893220,
  region: ES
})

/*=================================
      End of Code
==================================== */
```
