# Supplementary material for: Unveiling the ecology and spatial dynamics of Trypanosoma cruzi, its DTUs and Triatoma vitticeps in the Atlantic Forest of south-eastern Espírito Santo State, Brazil
Source: PLoS Negl Trop Dis. 2026 Mar 16;20(3):e0014111. doi: 10.1371/journal.pntd.0014111 (PMC13004508; doi:10.1371/journal.pntd.0014111)
Supplement: S2 Appendix — https://doi.org/10.1371/journal.pone.0143619. (PDF) [file pntd.0014111.s010.pdf]

```
/*==================================
      Beginning of Code
=================================== */

var dataset = ee.Image('CSP/ERGo/1_0/Global/SRTM_topoDiversity');
var srtmTopographicDiversity = dataset.select('constant').clip(ES);

var srtmTopographicDiversityVis = {
  min: 0.0,
  max: 1.0,
};
Map.setCenter(-111.313, 39.724, 6);
Map.addLayer(
    srtmTopographicDiversity, srtmTopographicDiversityVis,
    'SRTM Topographic Diversity');




Export.image.toDrive({
  image: srtmTopographicDiversity,
  description: 'TopographicDiversity_270m_Brasil',
  scale: srtmTopographicDiversity.projection().nominalScale().getInfo(),
  crs: 'EPSG: 4326',
  folder: 'EE',
  maxPixels: 49338893220,
  region: ES
})

/*==================================
        End of Code
=================================== */
```
